# Characterization of Genome-Wide Variation in Four-Row Wax, a Waxy Maize Landrace with a Reduced Kernel Row Phenotype

**DOI:** 10.3389/fpls.2016.00667

**Published:** 2016-05-18

**Authors:** Hanmei Liu, Xuewen Wang, Bin Wei, Yongbin Wang, Yinghong Liu, Junjie Zhang, Yufeng Hu, Guowu Yu, Jian Li, Zhanbin Xu, Yubi Huang

**Affiliations:** ^1^College of Life Science, Sichuan Agricultural UniversityYa’an, China; ^2^Germplasm Bank of Wild Species, Kunming Institute of Botany, Chinese Academy of Sciences, KunmingChina; ^3^Maize Research Institute, Sichuan Agricultural University, ChengduChina; ^4^College of Agronomy, Sichuan Agricultural University, ChengduChina; ^5^Seed Station of Xishuangbanna, JinghongChina

**Keywords:** Four-row Wax, genome-wide variations, SNPs, kernel row number, landraces of Chinese waxy maize

## Abstract

In southwest China, some maize landraces have long been isolated geographically, and have phenotypes that differ from those of widely grown cultivars. These landraces may harbor rich genetic variation responsible for those phenotypes. Four-row Wax is one such landrace, with four rows of kernels on the cob. We resequenced the genome of Four-row Wax, obtaining 50.46 Gb sequence at 21.87× coverage, then identified and characterized 3,252,194 SNPs, 213,181 short InDels (1–5 bp) and 39,631 structural variations (greater than 5 bp). Of those, 312,511 (9.6%) SNPs were novel compared to the most detailed haplotype map (HapMap) SNP database of maize. Characterization of variations in reported kernel row number (KRN) related genes and KRN QTL regions revealed potential causal mutations in *fea2, td1, kn1, and te1*. Genome-wide comparisons revealed abundant genetic variations in Four-row Wax, which may be associated with environmental adaptation. The sequence and SNP variations described here enrich genetic resources of maize, and provide guidance into study of seed numbers for crop yield improvement.

## Introduction

As the most widely grown C4 crop in the world, the first maize genome sequence was published in 2009 for a commercial variety called B73 (Schnable et al., 2009). Subsequently, much progress has been made to reveal genetic variation among maize varieties. A popcorn variety called *Palomero* was the second maize genome sequence published, and its comparison to B73 revealed variations, as well as monomorphic gene such as heavy metal detoxification genes that may have been selected during domestication (Vielle-Calzada et al., 2009). Additional genetic variations were detected from 27 inbred lines and a NAM population using NGS technology, and the resulting data was used in compiling the first generation maize HapMap (Gore et al., 2009; McMullen et al., 2009). The first HapMap revealed the genome-wide variation pattern of maize for the first time, although only ~38% of the maize genome was covered. Whole genome sequencing of 103 maize landraces and elite lines identified 55 million genome-wide SNPs (Chia et al., 2012; Hufford et al., 2012). Three types of variations including SNPs, SVs and genome size variations were included in the second generation maize HapMap (Chia et al., 2012). Characterization of the variations may help to suggest their roles and functions during maize domestication and improvement (Hufford et al., 2012). A growing collection of individual and population studies (Lai et al., 2010; Jiao et al., 2012; Xu et al., 2014) have also contributed to knowledge of maize variation and deducing functions of these variations. Despite these extensive studies of maize variation, sequencing of additional novel maize varieties can further enrich the variation dataset and may also benefit research into important traits found in the novel varieties.

**FIGURE 1:**
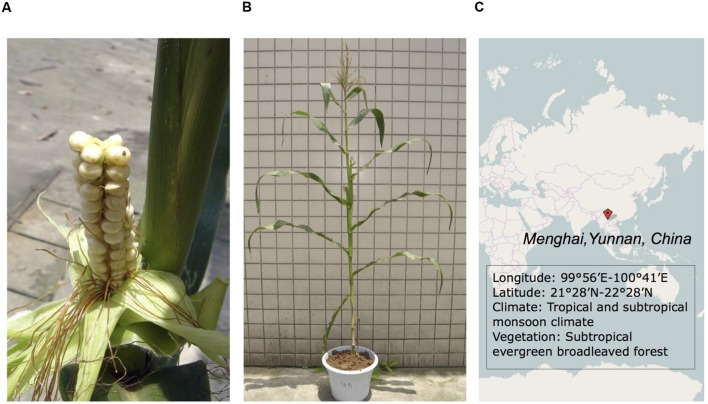
**Phenotypes of the Four-row Wax cob and plant. (A)** The cob with only four rows; **(B)** Four-row Wax, a common plant under normal cultivation management; **(C)** The geographic location and environmental conditions of Four-row Wax growth.

Waxy maize has a dramatic reduction in synthesis of amylose, caused by a recessive mutation of the waxy gene (*wx*; Wessler and Varagona, 1985; Zheng et al., 2013). Waxy maize may have originated in southwest China several 100 years ago (Tian et al., 2009). Four-row Wax is one of the waxy landraces, however, it has a special phenotype of only four rows of kernels on the cob (**Figures 1A,B**). It has a long history of production in Menghai County, Yunnan Province in southwest China as food by local Dai minority people (Tian et al., 2009). Four-row Wax is mainly grown in red latosol soils of mountainous areas, longitudes 99°56′E to 100°41′E and latitudes 21°28′N to 22°28′N (**Figure 1C**). This region has a tropical and subtropical monsoon climate, an annual average temperature of 18.7°C and total rainfall of 1341 mm. Four-row Wax is not known to have been subjected to scientific breeding (Tian et al., 2009). Researchers have been focused on this variety for some time, especially for its systematics and waxy trait (Fan et al., 2008, 2009; Tian et al., 2009). A 15-bp deletion at the 10th exon in *wx* is the mutation thought to be responsible for low amylose content in Four-row Wax (Fan et al., 2008, 2009). Phylogenetic analysis indicates that Four-row Wax is related to other Chinese waxy maize genotypes and landraces, in particular from neighboring Yunnan and Guangxi provinces, and suggests that Chinese waxy maize might have been derived from varieties of cultivated flint maize (Fan et al., 2008).

Both waxy endosperm and seed number are of importance for food quality and for crop improvement. Thus it is of interest and significance to analyze the genome of Four-row Wax for its genetic basis, domestication and evolution, to obtain diagnostic DNA markers for breeding, and also to provide clues to features of its features and those of other local maize varieties in China. Advances in NGS technologies make it possible to ‘resequence’ entire genomes efficiently and economically, providing the opportunity to identify and investigate genome-wide DNA polymorphisms, including SNPs, short InDels, SVs (Huang et al., 2010; Chia et al., 2012; Hufford et al., 2012; Xu et al., 2012; Romay et al., 2013). Further association of DNA-level variations with phenotypic differences will provide clues to interpret the genetic basis of important traits.

To gain insight into its genomic diversity, whole-genome resequencing of Four-row Wax was carried out using NGS technology. Genome-wide variations were identified through comprehensive bioinformatics comparisons. Analysis of novel variations and candidate genes were conducted in this paper with the aim of helping to explain the KRN trait of Four-row Wax. The data resources and information described here will increase our knowledge of maize diversity, help to speed up genetic dissection of KRN and guide future breeding applications to improve maize yield.

## Materials and Methods

### DNA Preparation and Sequencing

Four-row Wax, a Chinese waxy maize landrace, is not known to have been subjected to scientific breeding (Tian et al., 2009). We randomly selected a single individual from a traditional plot of this landrace with typical phenotypic characteristics, such as four rows of kernels on the cob, which was growing in the field with natural pollination at the flowering stage. We chose 10 open pollinated waxy seeds for genome sequencing by detecting KI/I2-staining of seeds and the 15-bp deletion mutation of *wx*. Thereafter, we ground 7-day-old etiolated seedlings in liquid nitrogen and extracted DNA using a CTAB (Hexadecyltrimethylammonium bromide)-based method, then randomly sheared the DNA. Fragments of ~500 bp were selected from a gel and used for a library preparation according to Illumina’s instructions. The libraries were sequenced at paired ends using an Illumina Hiseq 2000 in four sequencing lanes. The average length of sequencing reads was 90 bp.

The maize B73 reference genome sequence and gene set were downloaded from MaizeGDB (release version 4a.53; Schnable et al., 2009). We then filtered genes using the following criteria: (1) genes with CDS shorter than 150 bp were removed; (2) genes was discarded if a stop codon occurred within the coding region; (3) the longest transcript of a gene locus was retained to represent all transcripts at that locus; (4) overlapping genes were removed, retaining the longest one. After filtration, 75,747 genes were retained and were used for analysis.

### Assembly and Novel Gene Detection

*De novo* assembly was performed for all reads sequenced from Four-row Wax using SOAPdenovo software^1^ (version 1.05; Li et al., 2010). The k-mer parameter was set to 17. We then aligned the assembly against the B73 reference sequence. Regions which could not be mapped to the B73 genome were extracted and BLASTed against proteins of *Arabidopsis thaliana*, *Oryza sativa*, and *Sorghum bicolor* using default settings. Sequences with similarity higher than 70% to known proteins were considered as candidate novel genes.

### Short Read Mapping

We filtered low quality reads before mapping, including reads: (1) with more than 5% Ns or As; (2) with 20 bp or more having quality lower than 7; (3) contaminated by the adaptor (i.e., more than 10 bp aligned to the adaptor when allowing fewer than 3 bp mismatches); (4) with the first and second ends overlapping (10 bp aligned when allowing 10% mismatches); (5) with both ends identical to other reads, suggesting PCR duplication. The filtered reads were then aligned to the B73 reference genome sequence using SOAPaligner (version 2.18; Li et al., 2009b). We set the mapping parameter to allow at most two mismatches within one read. The sequencing depth and coverage were then calculated based on the alignment result. To detect small InDels, we conducted further mapping, setting the parameter ‘-g 5,’ which allowed less than or equal to 5 bp gaps.

### Identification of SNPs, InDels, and SVs

To detect SNPs, we used SOAPsnp software (version 1.03; Li et al., 2009a) using the following criteria: (1) The quality score given by SOAPsnp (which takes sequencing quality and ambiguity into consideration) should be greater than 20; (2) the sequencing depth should be higher than 2; (3) SNPs within 5 bp of nearby SNPs were considered as other variations; (4) SNPs with copy number greater than 1.5. SNPs meeting these filtering criteria were considered to be supported by high quality data, and to minimize possible errors caused by repeated sequences.

For short InDels’ the ‘gaps allowed’ mapping result above was subjected to InDel detection using SOAPindel (Li et al., 2013), and gaps supported by more than one third of the mapped reads were retained as qualified InDels. In this study, InDels are defined as insertions or deletions of 5 bp or less in length, while insertions or deletions of more than five nucleotides are classified as SVs.

The identified SNPs and InDels were classified based on occurrence in coding regions, non-coding regions, start codons, stop codons or splice sites. SNPs in coding sequences were annotated as synonymous or non-synonymous. GO or PFAM annotation of genes was also conducted.

Structural variations were detected using SOAPsv (version 1.02), for InDel (more than 5 bp in length), replication, reversion, transposition, and other variations. The minimum read depth was set to 3.

### Identification of Regions without SNPs

We first calculated the distances between adjacent SNPs, and the distribution of interval distance was depicted. Genes located between two adjacent SNPs were extracted as candidate genes without SNPs. Then, to further verify that there were no SNPs and other variations within those genes, we filtered the genes located in, or overlapping with, repeated elements (according to the annotation information), and genes that were not sequenced (no reads mapped to the gene region).

### Novel SNP Identification

We obtained the maize HapMap SNPs (in B73 RefGen_v1 coordinates) from the Panzea website (Chia et al., 2012). SNPs present in Four-row Wax (identified by mapping and aligning our reads to the B73 genome) but absent from the HapMap were considered novel.

### Variations Detection in Genomic Regions Associated with Kernel Row Number

We focused on four reported genes (Supplementary Table S10; Vollbrecht et al., 1991; Veit et al., 1998; Taguchi-Shiobara et al., 2001; Bommert et al., 2005, 2013; Lunde and Hake, 2009) and five large-effect QTLs (Supplementary Table S12; Yan et al., 2006; Li et al., 2007; Ma et al., 2007; Lu et al., 2010; Brown et al., 2011; Cook et al., 2012; Liu L. et al., 2015) related to KRN. We also compared variations in these regions between six elite inbred lines (Zheng58, 5003, 478, 178, Chang7-2 and Mo17) reported previously (Lai et al., 2010) and Four-row Wax.

## Results

### Resequencing, Mapping, and Assembly of the Four-Row Wax Genome

Using Four-row Wax genomic DNA, a total of 560.65 million sequencing reads of length 90 bp, totaling 50.46 Gb, were generated from four Illumina sequencing lanes (Supplementary Table S1). All sequence information was deposited at the Sequence Read Archive (SRA) of National Center for Biotechnology Information (NCBI), under accession number SRA090053. A total of 499.62 M reads (89.11%) were successfully mapped to the B73 maize reference genome (version: 4a.53) using software SOAP2. These reads covered 89.86% of the reference genome at average 21× depth.

In order to detect possible novel genes in Four-row Wax, we conducted a *de novo* genome assembly with software SOAPdenovo, and obtained a total assembled length of 996,022,706 bp (48.63% of the reference genome), believed to be mostly the low copy part of the maize genome. The contig N50 was 1022 bp, with the longest contig being 24,347 bp. The scaffold N50 was 2,410 bp, with the longest scaffold being 49,840 bp.

### Detection and Characterization of SNPs, InDels, and SVs

Sequencing depth affects accuracy of DNA variation detection (Arai-Kichise et al., 2011). Therefore, we compared the numbers of SNPs, InDels, and SVs detected from a set of combinations of resequencing data using different numbers of sequencing lanes, i.e., data with different coverages (Supplementary Table S1). The number of detected variations became stable above 10× coverage. Therefore, our 21.87× average depth of resequencing data is sufficient to identify genome-wide variations. A total of 3,252,194 SNPs, 213,181 InDels (1~5 bp), and 39,631 SVs were identified in the Four-row Wax genome as compared to the maize B73 reference sequence (**Table 1**).

**Table 1:** Distribution and density of SNPs, InDels, and SVs identified between Four-row Wax and B73 maize.

Chromosome	SNPs	SNPs/Mb	InDels	InDels/Mb	Ins	Ins/Mb	Del	Del/Mb	SV	SV/Mb
Chr1	473,967	1,579	32,998	110	15,324	51	17,674	59	5,960	20
Chr2	386,104	1,645	23,981	102	11,111	47	12,870	55	4,580	20
Chr3	364,401	1,581	24,643	107	11,335	49	13,308	58	4,310	19
Chr4	424,860	1,719	24,735	100	11,214	45	13,521	55	4,890	20
Chr5	313,126	1,444	22,491	104	10,269	47	12,222	56	4,019	19
Chr6	249,643	1,475	17,063	101	7,681	45	9,382	55	3,025	18
Chr7	287,446	1,681	17,952	105	8,185	48	9,767	57	3,360	20
Chr8	273,747	1,569	17,799	102	8,301	48	9,498	54	3,325	19
Chr9	237,802	1,561	16,279	107	7,508	49	8,771	58	2,984	20
Chr10	227,839	1,522	14,643	98	6,689	45	7,954	53	2,655	18
Chr unknown^1^	13,259	903	597	41	211	14	386	26	523	36
Total	3,252,194	1,578	213,181	103	97,828	47	115,353	56	39,631	19

*^1^Chr unknown indicates unplaced or unlocalized contigs.*

**FIGURE 2:**
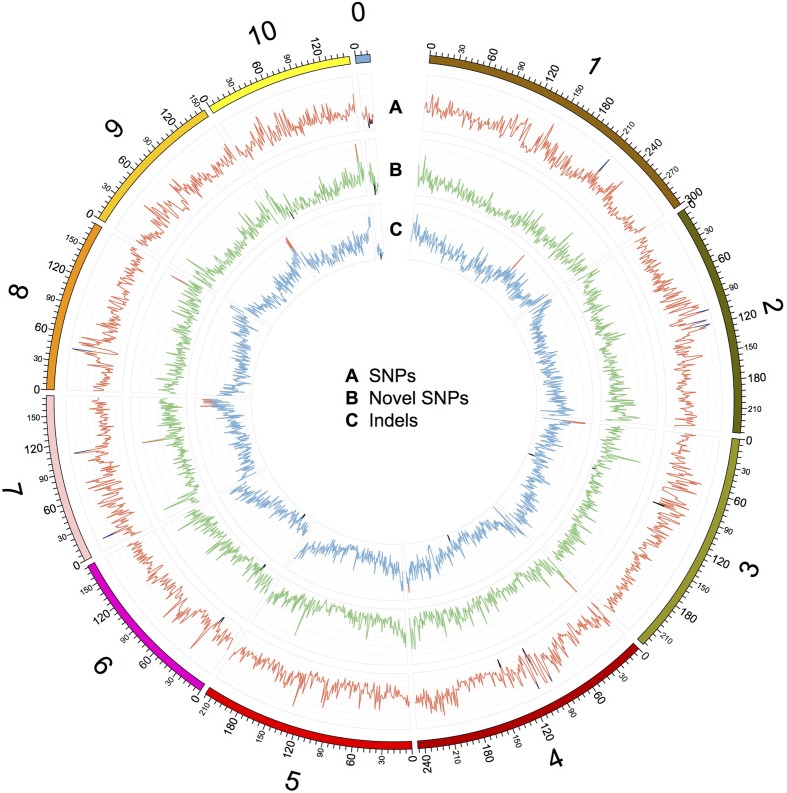
**Distribution of SNPs, novel SNPs, and InDels on the chromosomes of Four-row Wax.** The outer circle represents the physical distance along each chromosome, split into 1 Mb windows. A, B, and C indicate the density of SNPs, novel SNPs, and InDels, respectively. SNPs and InDels were identified by mapping Four-row Wax reads to the B73 genome, and novel SNPs were identified by comparing Four-row Wax SNPs with HapMap2. A: each grid denotes SNP density of 500/Mb, with line color indicating density >3500/Mb (blue), or <500/Mb (black). B: each grid denotes novel SNP density of 100/Mb, with line color indicating density >500/Mb (red) or <20/Mb (black). C: each grid denotes InDel density of 50/Mb, with line color indicating density >250/Mb (red) or <10/Mb (black). Chromosome 0 denotes sequences that are part of unplaced or unlocalized contigs.

**FIGURE 3:**
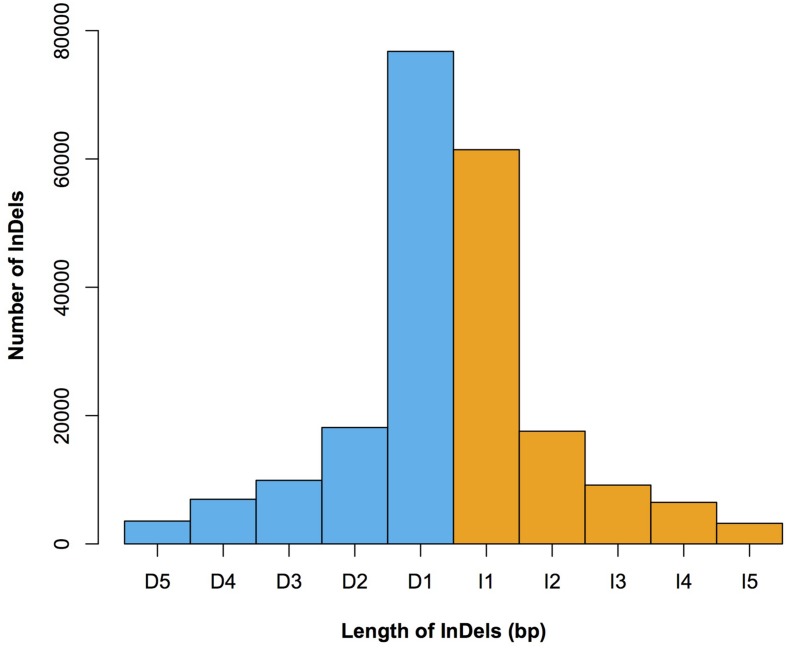
**The length distribution of 1–5 bp InDels in Four-row Wax.** Lengths (*x*-axis) and numbers (*y*-axis) of insertions (orange) and deletions (blue) are shown. I1–I5 denotes insertions of 1–5 bp in length, respectively; D1–D5 denotes deletions of 1–5 bp in length, respectively.

We characterized the distributions of SNPs, InDels, and SVs in the Four-row Wax genome (**Table 1**). The average densities of detected SNPs, InDels, and SVs were 1578, 103, and 19 per Mb, respectively. The density of SNPs or InDels was uneven among chromosomes (**Figure 2**). For SNPs, 10 and 9 regions with high (>3500/Mb) and low density (<500/Mb) were found, respectively. High SNP regions were on chromosomes 1, 2, 4, 7, and 8, while low SNP regions were on chromosomes 1, 2, 3, 4, 6, and 9. Interestingly, four of ten high SNP regions were on chromosome 2, the highest being 3962/Mb. Three of nine low SNP regions were on chromosome 4, the lowest being 181/Mb. There were eleven regions with high InDel density (>250/Mb) on chromosomes 1, 3, 4, 7, and 9, with the highest (285/Mb) on chromosome 9. Five regions with low InDel density (<10/Mb) were identified on chromosome 3, 4, and 6, the lowest (2/Mb) located on chromosome 4. The majority of InDels (63.87%) were mononucleotide variations, including 61,433 insertions and 76,778 deletions (**Figure 3**). The abundance of InDels decreased with increasing length, while different InDels of similar length had similar abundance.

**FIGURE 4:**
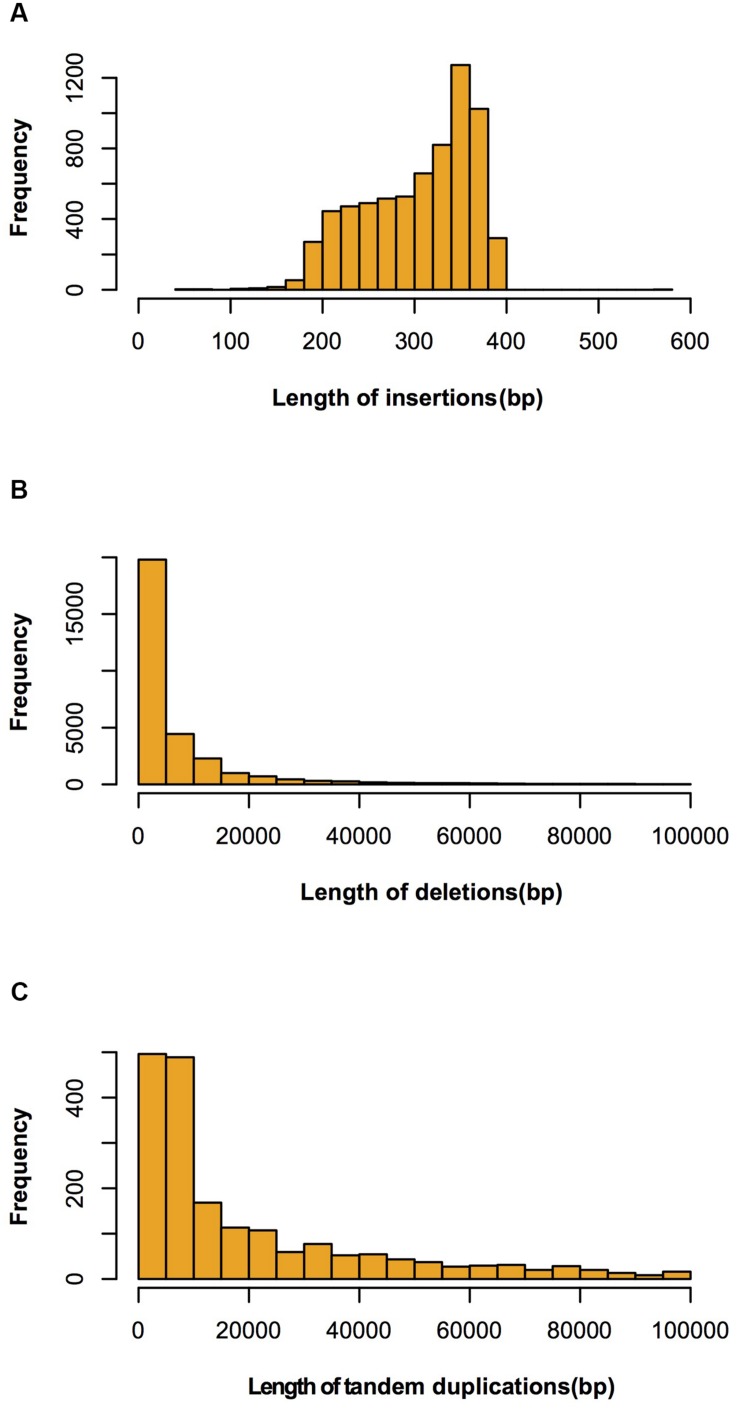
**Length distributions of large insertions, deletions and tandem duplications identified between Four-row Wax and B73. (A)** insertions; **(B)** deletions; **(C)** tandem duplications.

Four regions had high SV density (>80/Mb) on chromosomes 1, 4, and 8. The highest SV density (145/Mb) region was on chromosome 1 and the lowest (1/Mb) was on chromosome 4. The average SV density on each chromosome was similar, although SV variations were not uniformly distributed within a chromosome (**Table 1**). Of the 39,631 SVs, 30,046, 6,879, and 1,887 were classified as deletion type, insertion type and tandem duplication, respectively. The types, numbers and length distributions of SVs are shown in **Figure 4**.

### Annotation of SNPs and InDels in Four-Row Wax

We annotated the sequences containing detected SNPs and InDels according to the gene annotation of the B73 reference genome (Schnable et al., 2009). Only 587,303 SNPs (18.06%) and 63,927 InDels (29.99%) were located in genic regions, the majority being in intergenic regions. The number of SNPs and InDels in gene coding sequences (CDSs), introns and UTRs are shown in **Table 2**, and their distributions on each chromosome are in Supplementary Tables S2 and S3. Of those variations in genic regions, 26.15% SNPs and 5.95% InDels were in CDSs while the remainder were in introns. Of those in CDSs, 46.36% of SNPs were non-synonymous, and 62.36% of InDels induced translation frame-shifts, while the remaining InDels (1,431) caused deletion/insertion of amino acids.

**Table 2:** Annotation of SNPs and InDels identified between Four-row Wax and B73 maize.

Variation types	Total	Intergenic	Genic	Intron	UTR^1^	CDS^2^	Non-Synonymous/Frame_shift	Synonymous/X3_shift
SNPs	3,252,194	2,664,891	587,303	314,146	119,588	153,569	71,198	82,371
InDels	213,181	149,254	63,927	42,556	17,569	3,802	2,371	1,431

*^1^UTR is the untranslated region of exons. ^2^CDS is coding DNA sequence, composed of exons coding for protein.*

We further classified the non-synonymous SNPs for inferred effects on gene function (**Table 3**). Of all non-synonymous SNPs, 2,617 located in 2,259 transcripts induced premature stop codons; 681 abolished stop codons, resulting in longer open reading frames; 298 altered initiation methionine residues. A total of 2,769 SNPs were located within splice sites (1–3 bp into exons or 2 bp into introns), 718 of which affected essential splice donor or acceptor sites (first 2 bp or last 2 bp of an intron), resulting in 704 transcripts with open reading frame changes. In addition, 48,523 SNPs, located in 21,663 transcripts, encoded amino acids with significant property difference from those of B73, based on four types of hydrophobic, negative, positive, and polar without charge properties.

**Table 3:** Types and numbers of large effect SNPs identified between Four-row Wax and B73 maize.

Types	Total number	Inducing premature stop codons	Abolishing stop codons	Altering initiation Met	Altering splice sites	Essential splice sites	Changing properties of amino acids
Non-synonymous of total SNPs	71,198	2,617 (3.68%)	681 (0.96%)	298 (0.42%)	2,769 (3.89%)	718 (1.01%)	48,523 (68.15%)
Non-synonymous of novel SNPs	23,590	1,129 (4.79%)	187 (0.79%)	76 (0.32%)	952 (4.04%)	358 (1.52%)	14,465 (61.32%)

A total of 22,264 transcripts were predicted to contain ‘large-effect’ SNPs, which changed start codons, stop codons, splice sites or amino acids encoded (**Table 3**). GO analysis (Supplementary Table S4 and Supplementary Figure S1) showed that large-effect SNPs were significantly enriched in genes encoding serine/threonine kinase, tyrosine kinase, nucleoside-triphosphatase, serine hydrolase, and binding proteins, such as ATP binding, sugar binding, and heat shock protein.

### Regions without SNPs

We calculated the distance between each adjacent pair of SNPs, and found many with short intervening distances (Supplementary Figure S2). The distance between 1,793,165 adjacent SNPs (55.14%) was shorter than 100 bp, while 1,102,673 adjacent SNPs (33.9%) were more than 100 bp but less than 1 kb apart. Only 10.96% of adjacent SNPs are 1 kb or more apart. Interestingly, we identified 39,865 regions of more than 10 kb without SNPs, i.e., which have identical sequences between B73 and Four-row Wax. A total of 28,952 genes were located in these invariant regions, including many encoding proteins with important biological functions essential for life, and mainly participating in primary metabolic processes (Supplementary Table S5 and Supplementary Figure S3), such as ligand-dependent nuclear receptor, electron carrier and enzymes with catalytic activity (NADH dehydrogenase, protein disulfide oxidoreductase, cysteine-type peptidase, and aspartic-type endopeptidase). Additionally, some proteins with DNA binding and metal ion (copper or zinc ion) binding activity were also enriched.

### Detection of Novel Variations and Novel Gene Candidates

Specific variations in an individual may explain its unique phenotypes. We further analyzed novel variations in Four-row Wax, finding that 2,043,063 (62.8%) of the 3,252,194 SNPs differentiating it from B73 can also be found in the most comprehensive genome-wide maize SNP dataset, maize HapMap (Chia et al., 2012), while the remaining SNPs are unique to Four-row Wax. After filtering out SNPs in repetitive regions of the maize genome where SNP detection may be unreliable, 312,511 (9.6%) novel SNPs were identified in non-repetitive regions. The chromosome distributions and annotations of those novel SNPs were analyzed and listed in Supplementary Table S6. The average density of novel SNPs on each chromosome was similar, but there were high-density or low-density regions (**Figure 2**). Four regions with high novel SNP density (>500/Mb) were located on chromosomes 4, 7, 8, and 10, respectively. Seven regions with low novel SNP density (<20/Mb) were located on chromosomes 3, 4, 6, 9, and 10, respectively. Comparing the distributions of SNPs and InDels, we found some overlapping regions, such as a region (located about 216M of chromosome 1) with high SNPs and InDels; two regions (~90M of chr 3 and ~138M of chr 4) with low SNPs and InDels; and two regions (~27M of chr 6 and ~7M of chr 0) with low densities of all three kinds of variations (SNPs, novel SNPs, and InDels; **Figure 2**). Most novel SNPs, 248,518 (79.52%), were located in intergenic regions and introns; with 37,198 (11.9% of) novel SNPs in coding sequences. The distribution of novel SNPs was similar to that of total detected SNPs described above. Novel SNPs were distributed in 24,285 genes, mainly in introns and UTRs, with only a few in CDSs. Among the ten genes containing the highest number of novel SNPs (Supplementary Table S7), six were predicted to encode binding proteins, including proteins binding zinc ions, DNA or ATP. Three genes have known functions encoding GTPase, tyrosine protein kinase and pentatricopeptide.

Similar to total SNPs, 63.42% of novel SNPs in Four-row Wax were non-synonymous. Of those, 16,809 (71.25%) belonged to the large effect SNP class. The types and numbers of large effect SNPs are provided (**Table 3**). Among all SNPs, the number that were non-synonymous was far more than the number of novel SNPs, so the large effect SNP number for each type was more than that of novel SNPs. However, for total SNPs and novel SNPs, the proportions that were non-synonymous were similar. Two types of mutations (premature stop codons and essential splice sites) occurred at higher proportions in novel SNPs than total SNPs. GO term analysis revealed that of genes enriched for these large effect novel SNPs were in four categories based on molecular function (Supplementary Table S8 and Supplementary Figure S4), including genes encoding ATP binding proteins, nucleoside-triphosphatase, serine/threonine kinase, and tyrosine kinase.

In the current maize database, InDels were not well documented and SVs were not accurate due to short reads and low depth of sequencing, so novel InDels and SVs in Four-row Wax were not predicted in this study.

The phenotypes that are characteristic of Four-row Wax may be caused by large sequence variations such as novel genes. We annotated the assembled Four-row Wax genome to seek novel genes, selecting scaffolds longer than 1 Kb and without corresponding annotation information in the reference genome. By comparison to gene sets of *Arabidopsis*, rice and sorghum, 583 novel genes were predicted, of which 123 have homologs in the current plant protein database (Uniprot and KEGG). Four novel maize genes were identified based on less than 20% similarity to other genes in the maize protein database (Supplementary Table S9), encoding a zinc transporter 2, pentatricopeptide repeat-containing protein, phloem-specific lectin and saposin-like type B, respectively. Such striking divergence from other maize genes makes these of interest to study further as potential contributors to phenotypic differences between Four-row Wax and other maize genotypes.

### Variations in the Regions Associated with KRN

Kernel row number is an important trait for maize yield. Previous research showed that it was a quantitative trait controlled by multiple genes, each with small phenotypic effects (Lu et al., 2010). Several QTLs and known genes have been associated with KRN (Lu et al., 2010; Brown et al., 2011; Cook et al., 2012; Bommert et al., 2013). We examined variations in Four-row Wax for four known KRN related genes (Supplementary Table S10), finding no SVs or novel SNPs. Some InDels and SNPs (all synonymous) were detected but were distributed in CDS, intron or 3’UTR regions (Supplementary Table S11). An InDel caused by a 3-bp deletion of AGG in the CDS of terminal ear1 (*te1*), resulted in the fourth position deletion of five consecutive glutamic acids, which may alter gene function and contribute to the low KRN phenotype in Four-row Wax.

**FIGURE 5:**
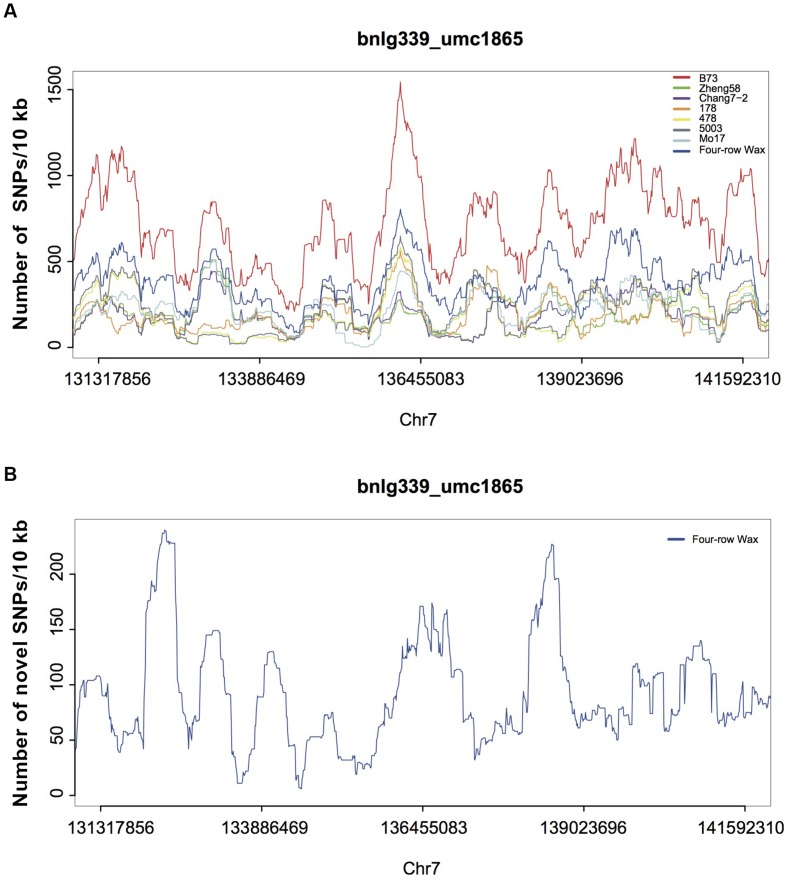
**SNPs densities in maize genomic regions associated with KRN (on chromosome 7). (A)** SNP density distributions for Four-row Wax, B73 and 6 elite inbred lines, in 10 kb windows throughout one published large-effect QTL region (on chromosome 7). The sum of SNP densities for the other six lines is plotted along the B73 reference genome. **(B)** Novel SNPs density of the QTL region for Four-row Wax.

To further investigate possible variations responsible for KRN in maize, we analyzed five large-effect QTLs (Supplementary Table S12; Yan et al., 2006; Li et al., 2007; Ma et al., 2007; Lu et al., 2010; Brown et al., 2011; Cook et al., 2012), accounting for high proportions of the KRN phenotype. DNA variations in Four-row Wax for the five QTL regions are listed in Supplementary Table S13. The average density of each variation in the QTL regions was similar to that in the whole genome. The reported QTLs, obtained from linkage mapping, cover large genomic regions while only one or a few sites may cause the KRN phenotype. Thus, we investigated patterns of variation in smaller windows, of 10 Kb. We also compared the SNP densities with those of six previously published elite inbred lines (Lai et al., 2010), which had significant KRN differences and are widely used in Chinese maize breeding (**Figure 5** and Supplementary Figure S6). SNP densities in a few 10 kb regions were substantially high in Four-row Wax than other genotypes. For each QTL, the highest SNP-density region in Four-row Wax was scrutinized as a potential causal region of low KRN. There are a total of 88 genes (Supplementary Table S14) in the five QTL regions, of which 52 were functionally known. Among the known genes, we found one auxin responsive gene called small auxin up-regulated RNA (SAUR), for which family members were reported to reduce growth and seed yield, such as *SAUR39* in rice (Kant and Rothstein, 2009; Kant et al., 2009). Whether the SAUR gene may affect KRN in Four-row Wax is worthy of further investigation.

## Discussion

### Rich Variation in the Four-Row Wax Genome

Modern maize has experienced domestication as a result of selection from *Zea mays* ssp. Parviglumis, and subsequent genetic improvement. A single domestication event is estimated to have taken place between 6000 and 9000 years ago in southern Mexico (Matsuoka et al., 2002), which resulted in the original maize landrace varieties. In the 20th century, breeders selected maize varieties and inbred lines from landraces for hybrid production, improving yield, resistance to biotic and abiotic stresses and seed nutritional quality (Jiao et al., 2012; Liu H. et al., 2015). Glutinous maize was first recorded in China in 1909, and was believed to have arisen from improvement of domesticated non-glutinous maize (Fan et al., 2008). Most Chinese waxy maize landraces maintained rich genetic diversity, with different genetic improvement processes from modern improved maize lines (Fan et al., 2009). Some landraces had phenotypic variations in important agronomic traits, and were helpful to identify and characterize genes responsible for those traits. Using this knowledge, plant breeders can further improve crop varieties.

With rapid development of sequencing technologies, maize genome sequencing studies have been widely undertaken. Building on the completion of the B73 reference genome sequence (Schnable et al., 2009), resequencing of a number of maize lines (Chia et al., 2012; Hufford et al., 2012; Romay et al., 2013) provided opportunities to unravel the tremendous genetic variation and diversity of maize at the whole genome scale. DNA polymorphisms among maize lines could be utilized for genetic analysis as well as crop improvement.

In our study, the maize landrace called Four-row Wax was resequenced and mapped to the B73 reference genome to discover genome-wide DNA variations. We uncovered more than three million SNPs and InDels, along with large numbers of SVs. Six resequenced elite maize inbred lines (Lai et al., 2010) include the parents of the most productive commercial hybrids in China and the USA, and are representatives of the main heterotic groups (B73, 478, 5003 and Zheng58 are in the Reid heterotic group; Mo17 and 178 are in the Lancaster heterotic group; Chang7-2 is in the Tangsipingtou group). Compared to the six inbred lines, Four-row Wax was much richer in SNPs and InDels. The abundance of variation in Four-row Wax may be partly related to higher sequencing depth (21.87×) in our data than in those inbred lines (5.4×). However, the greater number of SNPs and InDels in Four-row Wax may also reflect greater genetic diversity. A phylogenetic tree constructed from identified SNPs (Supplementary Figure S5), showed Four-row Wax to be more distant from B73 than the elite maize inbreds. A relatively close genetic relationship was found between Four-row Wax and Chang7-2, with a more distant relationship to the other 5 lines and B73. Huangzaosi is an important inbred line widely used in China, derived from landraces in Huanghuaihai area. Chang7-2 is a line derived from Huangzaosi, and it has become a member of the important Tangsipingtou heterotic group used in China. This finding shows that Four-row Wax is more closely related to the Tangsipingtou group originated from Huanghuaihai area of China than to members of the important Reid or Lancaster heterotic groups. The results of phylogenetic analysis are consistent with a previous report of the relationships and characteristics of inbred lines (Lai et al., 2010), supporting the validity of genome resequencing and variation identification.

Chia et al. (2012) identified 55 million SNPs in 103 inbred lines representing a wide breadth of the *Z. mays* lineage, including 60 improved lines, 23 landraces, and 19 wild relatives, which are resources in the most comprehensive maize SNP datasets currently available, HapMap2. Most SNPs detected in Four-row Wax were also found in the HapMap2 dataset, but 312,511 (9.6% of) SNPs were unique to Four-row Wax, excluding 774,231 (23.81%) SNPs in repeated regions. The new SNPs detected in Four-row wax enrich the current HapMap database. Novel SNPs in Four-row Wax may have resulted from the following scenarios: (1) Four-row Wax was a landrace, derived from southwestern China, with a different genetic improvement history from modern maize lines; (2) maize is a cross-pollinated plant harboring rich genetic diversity, especially among different landraces.

### Genes with or without Abundant Variation

We found that protein kinases (such as serine/threonine kinase and tyrosine kinase) were enriched for large-effect SNPs. This tendency was also found in previous resequencing in maize (Lai et al., 2010) and other species, such as rice (McNally et al., 2009), sorghum (Zheng et al., 2011), and *Arabidopsis* (Clark et al., 2007). It was reported that these kinases participated in responses to stresses such as drought, high or low temperature; and plant diseases and pests. To adapt to new environments and improve resistance to pests, plant stress-resistance genes may commonly experience diversifying selection. Among novel large-effect SNPs in Four-row Wax, we identified enrichment in genes related to biotic and abiotic stress, such as pathogenesis-related transcription factor, tyrosine protein kinase and armadillo-type fold. Novel SNPs in these Four-row Wax genes may result from the selection and adaptation to local environments.

In the Palomero landrace (Vielle-Calzada et al., 2009), genes involved in heavy-metal detoxification have low nucleotide variability, such as the cadmium transporter, a P-type copper translocator and a phosphatidylinositol transporter. A similar result was found in Four-row Wax maize, as proteins with metal ion binding activity were enriched in conservative genomic regions, without SNPs. On one hand, proteins binding metal ions generally possess transcriptional regulator activity or catalytic activity, and participate in basic metabolic processes (Berg, 1986). Meanwhile, these proteins may be involved in metal transport for heavy-metal detoxification. In partial summary, our results are similar to those based on resequencing of Mexican landrace Palomero.

Novel traits in Four-row Wax may also be caused by novel genes. We *de novo* assembled the genome sequence using 21.87× coverage reads, though sequence from only one insert size library was not enough to fully assemble the genome. Only four novel maize genes were predicted in the limited assembled sequence, which did not exist in the reference genome and were not homologous to annotated genes. One gene is homologous to a rice gene, and the remaining three are homologous to sorghum genes. The four genes showed the same overall depth of sequence coverage as other annotated genes, making it unlikely that these reads were due to contaminant DNA. The four novel genes may be really present only in the genome of Four-row Wax but not in other maize lines, because the genome of maize is complex and diversifying, with PAVs well known among genomes (Lai et al., 2010). We cannot rule out the possibility that the four genes exist in B73 and other maize genomes but were not included in the current B73 assembly, or not identified in other maize lines by molecular biology experiments. However, the chance is very low.

### Potential Variations for Low KRN

Low KRN is a characteristic phenotypic trait of Four-row Wax, making this genotype a valuable resource to investigate KRN. The ancestor of maize, teosinte, makes two rows of kernels while modern varieties and inbred lines have 10–20 rows (Bommert et al., 2013). Fewer kernel rows leads to lower yield, thus KRN is an important trait that human selection has altered during domestication and genetic improvement. Many studies have identified QTLs for maize KRN (Lu et al., 2010; Brown et al., 2011; Cook et al., 2012) but only a few genes have been isolated by map-based cloning or mutant analysis (Vollbrecht et al., 1991; Bommert et al., 2005, 2013), and the molecular mechanism underlying this trait is largely unknown. QTL for inflorescence traits have been discovered by both joint linkage mapping and GWAS in a maize NAM population (Brown et al., 2011). QTL for grain yield components involved in early maize domestication have been found in a population of maize-teosinte BC2S3 RILs (Shannon, 2013), segregating for allelic variation not present in the NAM population. Based on the two maize populations, some QTL associated with KRN were identified. Additionally, a few genes affecting KRN and required for shoot meristem establishment, maintenance and size, have been isolated (Supplementary Table S10). Among them, *fea1* and *fea2* cause massive over-proliferation of the ear inflorescence meristem and lead to increased KRN, but a mutation of *kn1* displayed small ears, with length, diameter and KRN smaller than normal plants (Bommert et al., 2005).

In the CDS of the four genes previously implicated in genetic control of KRN, *fea2*, *td1*, *kn1*, and *te1*, all SNPs in Four-row Wax were synonymous. However, one InDel caused loss of a glutamic acid residue in the C terminus of te1 although the InDel is not in a conserved domain (Supplementary Table S11). Clarifying the function of the InDels in *te1* needs further molecular and physiological investigation *in vivo*. Bommert et al. (2013) sequenced the *fea2* locus and compared Mo17 and B73, finding no non-synonymous changes, although B73 had significantly higher KRN and a larger inflorescence meristem than Mo17. Hence, changes in the fea2 protein sequence are not responsible for variation in KRN between B73 and Mo17. However, *fea2* expression from the B73 allele was threefold lower than that of the Mo17 allele as revealed by RT-PCR, likely due to regulatory changes in the *fea2* alleles. Likewise, we inferred that SNPs and InDels in the genic region of the gene may interact with additional genes together to alter the KRN. Li et al. (2012) reported that trait-associated SNPs were enriched in the non-genic regions, particularly within a 5-kb window upstream of genes. To get further information, we investigated variation within 5 kb upstream of the four genes in Four-row Wax (Supplementary Table S11), finding that *fea2* and *td1* had more variation than *kn1* and *te1*. Some of these variations may contribute to the low KRN of Four-row Wax.

Besides the above four genes, *ids1* (Chuck et al., 1998), indeterminate spikelet 1, specifies a determinate spikelet meristem fate and thereby limits the number of floral meristems produced. In the absence of *ids1* gene function, the spikelet meristem becomes indeterminate and produces additional florets. So KRN increased in the *ids1* mutation. However, in the annotation of the reference genome version 1 (4a.53), we could not find the gene, thus could not analyze variation in it.

Moreover, we compared DNA polymorphisms between Four-row Wax and six inbred lines in five QTLs related to KRN. SNPs, InDels, and SVs in these regions may be responsible for low KRN. Because the reported QTL regions delimited by linked molecular markers were large, with many differences among compared varieties, the causal DNA polymorphisms contributing to low KRN have not been identified. Some genes located in these regions are enriched in novel SNPs and identified as potential candidate genes for the low KRN of Four-row Wax, such as the auxin responsive gene of SAUR. It is of high priority to investigate the relationship between these genes and the KRN phenotype.

### Conclusion

In summary, we identified 3.3 M SNPs, 213.2 K InDels, and 39.6 K SVs distinguishing Four-row Wax, a waxy maize landrace with low KRN from southwestern China, from the B73 reference genome. This variety harbored novel SNPs (absent from the present maize Hapmap), and novel genes were also identified by comparing to known maize variation or gene datasets. By analyzing the Four-row Wax variations in reported genes and QTLs related to KRN, candidate genes and mutations related to its low KRN phenotype were inferred. These data and results, combined with other variation and population studies of maize, may facilitate the genetic analysis and improvement of important agronomic traits of maize.

## Author Contributions

YH and HL conceived and designed the experiments. YL, JL, and ZX contributed materials. BW, HL, JZ, and GY contributed to data analysis. YW and YH performed the experiments. HL and XW wrote and revised the manuscript. All authors discussed the results and commented on the manuscript.

## Conflict of Interest Statement

The authors declare that the research was conducted in the absence of any commercial or financial relationships that could be construed as a potential conflict of interest.

## Footnotes

1 http://soap.genomics.org.cn

## Abbreviations

GO: gene ontology
HapMap: haplotype map
InDel: insertion and deletion
KRN: kernel row number
NAM: nested association mapping
NGS: next generation sequencing
PAV: presence/absence variation
SNP: single nucleotide polymorphism
SV: structure variation
UTR: untranslated region.
