# Supplementary material for: Characterization of Genome-Wide Variation in Four-Row Wax, a Waxy Maize Landrace with a Reduced Kernel Row Phenotype
Source: Front Plant Sci. 2016 May 18;7:667. doi: 10.3389/fpls.2016.00667 (PMC4870249; doi:10.3389/fpls.2016.00667)
Supplement: Supplementary file 1 [file Table_1.DOC]

# *Supplementary Material*

**Characterization of genome-wide variations in Four-row Wax, a waxy maize landrace with a reduced kernel row phenotype**

**Hanmei Liu^1†^, Xuewen Wang^5†^, Bin Wei^2†^, Yongbin Wang^2^, Yinghong Liu^2^, Junjie Zhang^1^, Yufeng Hu^3^, Guowu Yu^3^, Jian Li^4^, Zhanbin Xu^4^, Yubi Huang^2,3^,***

***Correspondence:** Yubi Huang, [yubihuang@sohu.com](mailto:yubihuang@sohu.com)

# Additional file 2: Supplementary tables S1 to S13

**Table S1: Relationships among sequencing depth, output data and the number of detected DNA polymorphisms.**

**Table S2: Annotation of SNPs on each chromosome.**

**Table S3: Annotation of InDels on each chromosome.**

**Table S4: GO enrichment of genes with large effect SNPs.**

**Table S5: GO enrichment of genes without SNPs.**

**Table S6: The distribution and annotation of novel SNPs.**

**Table S7: The ten genes most enriched in novel SNPs.**

**Table S8: GO enrichment of genes with novel large effect SNPs.**

**Table S9: Four novel genes in Four-row Wax.**

**Table S10: Isolated genes contributing to changing KRN.**

**Table S11:Variation in genes contributing to changing KRN, in Four-row Wax.**

**Table S12: Five large effect QTLs contributing to changing KRN.**

**Table S13: Variations in five QTL regions in Four-row Wax.**

**Table S14: Potential causal genes of the reduced kernel row trait in Four-row Wax.**

# ****Supplementary Tables****

**Supplementary Table 1** Relationships among sequencing depth, output data and the number of detected DNA polymorphisms

| Lane | Depth | Reads(M) | Bases(G) | Mapped Reads(M) | Mapped Bases(G) | Coverage (%) | SNP | InDel | SV |
| --- | --- | --- | --- | --- | --- | --- | --- | --- | --- |
| 1 | 5.31 | 136.22 | 12.26 | 121.40 8 | 10.88 | 79.36 | 618,809 | 104,065 | 19,856 |
| 2 | 10.83 | 277.65 | 24.99 | 247.34 8 | 22.18 | 85.89 | 1,903,988 | 174,488 | 34,894 |
| 3 | 16.31 | 418.29 | 37.65 | 372.51 8 | 33.4 | 88.38 | 2,702,280 | 202,012 | 37,257 |
| 4 | 21.87 | 560.65 | 50.46 | 499.62 8 | 44.79 | 89.86 | 3,252,194 | 213,181 | 39,631 |

**Supplementary Table 2** Annotation of SNPs on each chromosome

| Chromosome | Sum | Intergenic | Genic | CDS | | | 5'UTR | 3'UTR | Intron |
| --- | --- | --- | --- | --- | --- | --- | --- | --- | --- |
|  |  |  |  | Total | Non-syn | Syn |  |  |  |
| chr1 | 473,967 | 382,030 | 91,937 | 23,110 | 10,936 | 12,174 | 7,643 | 10,196 | 50,988 |
| chr2 | 386,104 | 313,479 | 72,625 | 18,926 | 8,765 | 10,161 | 6,063 | 8,942 | 38,694 |
| chr3 | 364,401 | 301,159 | 63,242 | 16,780 | 7,680 | 9,100 | 5,225 | 7,612 | 33,625 |
| chr4 | 424,860 | 363,321 | 61,539 | 16,178 | 7,198 | 8,980 | 5,612 | 7,086 | 32,663 |
| chr5 | 313,126 | 249,476 | 63,650 | 16,435 | 7,873 | 8,562 | 5,276 | 7,891 | 34,048 |
| chr6 | 249,643 | 202,253 | 47,390 | 12,830 | 5,913 | 6,917 | 4,582 | 5,669 | 24,309 |
| chr7 | 287,446 | 236,851 | 50,595 | 13,035 | 6,242 | 6,793 | 4,252 | 5,962 | 27,346 |
| chr8 | 273,747 | 223,089 | 50,658 | 13,099 | 5,956 | 7,143 | 4,168 | 6,265 | 27,126 |
| chr9 | 237,802 | 192,678 | 45,124 | 11,642 | 5,432 | 6,210 | 3,789 | 5,108 | 24,585 |
| chr10 | 227,839 | 188,390 | 39,449 | 11,271 | 5,093 | 6,178 | 3,392 | 4,617 | 20,169 |
| Chr unknown | 13,259 | 12,165 | 1,094 | 263 | 110 | 153 | 106 | 132 | 593 |
| Total | 3,252,194 | 2,664,891 | 587,303 | 153,569 | 71,198 | 82,371 | 50,108 | 69,480 | 314,146 |

**Supplementary Table 3** Annotation of InDels on each chromosome

| Chromosome | Sum | Intergenic | Genic | CDS | | | 5'UTR | 3'UTR | Intron |
| --- | --- | --- | --- | --- | --- | --- | --- | --- | --- |
|  |  |  |  | Total | Triple | Shift |  |  |  |
| chr1 | 32,998 | 22,848 | 10,150 | 542 | 191 | 351 | 1,029 | 1,661 | 6,918 |
| chr2 | 23,981 | 16,622 | 7,359 | 444 | 189 | 255 | 731 | 1,200 | 4,984 |
| chr3 | 24,643 | 17,524 | 7,119 | 420 | 140 | 280 | 740 | 1,264 | 4,695 |
| chr4 | 24,735 | 18,082 | 6,653 | 387 | 146 | 241 | 733 | 1,132 | 4,401 |
| chr5 | 22,491 | 15,212 | 7,279 | 400 | 149 | 251 | 812 | 1,270 | 4,797 |
| chr6 | 17,063 | 11,863 | 5,200 | 337 | 140 | 197 | 643 | 914 | 3,306 |
| chr7 | 17,952 | 12,570 | 5,382 | 318 | 125 | 193 | 578 | 880 | 3,606 |
| chr8 | 17,799 | 12,169 | 5,630 | 340 | 126 | 214 | 548 | 1,020 | 3,722 |
| chr9 | 16,279 | 11,408 | 4,871 | 309 | 107 | 202 | 531 | 743 | 3,288 |
| chr10 | 14,643 | 10,455 | 4,188 | 299 | 114 | 185 | 439 | 679 | 2,771 |
| Chr unknown | 597 | 501 | 96 | 6 | 4 | 2 | 9 | 13 | 68 |
| Total | 213,181 | 149,254 | 63,927 | 3802 | 1431 | 2371 | 6,793 | 10,776 | 42,556 |

****Supplementary Table 4 GO enrichment of genes with large effect SNPs****

| GO-ID | Description | Cluster freq | Total freq | P-value | Coor P-value |
| --- | --- | --- | --- | --- | --- |
| GO:001882 | nucleoside binding | 1462/8263 | 4900/36469 | 2.28E-36 | 2.50E-33 |
| GO:030554 | adenyl nucleotide binding | 1461/8263 | 4898/36469 | 2.83E-36 | 2.50E-33 |
| GO:001883 | purine nucleoside binding | 1461/8263 | 4898/36469 | 2.83E-36 | 2.50E-33 |
| GO:032559 | adenyl ribonucleotide binding | 1387/8263 | 4652/36469 | 3.68E-34 | 2.44E-31 |
| GO:005524 | ATP binding | 1384/8263 | 4648/36469 | 8.69E-34 | 4.60E-31 |
| GO:000166 | nucleotide binding | 1679/8263 | 5848/36469 | 2.35E-32 | 1.04E-29 |
| GO:017076 | purine nucleotide binding | 1594/8263 | 5535/36469 | 3.13E-31 | 1.18E-28 |
| GO:032555 | purine ribonucleotide binding | 1517/8263 | 5277/36469 | 3.63E-29 | 1.07E-26 |
| GO:032553 | ribonucleotide binding | 1517/8263 | 5277/36469 | 3.63E-29 | 1.07E-26 |
| GO:003824 | catalytic activity | 4128/8263 | 16585/36469 | 8.84E-21 | 2.34E-18 |
| GO:016773 | phosphotransferase activity, alcohol group as acceptor | 783/8263 | 2614/36469 | 1.76E-19 | 4.23E-17 |
| GO:005488 | binding | 5456/8263 | 22606/36469 | 2.79E-18 | 6.17E-16 |
| GO:016740 | transferase activity | 1433/8263 | 5242/36469 | 5.11E-18 | 1.04E-15 |
| GO:005515 | protein binding | 1188/8263 | 4266/36469 | 1.85E-17 | 3.51E-15 |
| GO:004672 | protein kinase activity | 669/8263 | 2231/36469 | 7.76E-17 | 1.36E-14 |
| GO:016772 | transferase activity, transferring phosphorus-containing groups | 949/8263 | 3328/36469 | 8.20E-17 | 1.36E-14 |
| GO:004674 | protein serine/threonine kinase activity | 618/8263 | 2047/36469 | 2.88E-16 | 4.49E-14 |
| GO:006468 | protein amino acid phosphorylation | 654/8263 | 2188/36469 | 3.75E-16 | 5.52E-14 |
| GO:016301 | kinase activity | 803/8263 | 2770/36469 | 4.39E-16 | 6.11E-14 |
| GO:004713 | protein tyrosine kinase activity | 570/8263 | 1878/36469 | 1.49E-15 | 1.98E-13 |
| GO:012501 | programmed cell death | 74/8263 | 143/36469 | 3.13E-14 | 3.77E-12 |
| GO:006915 | apoptosis | 74/8263 | 143/36469 | 3.13E-14 | 3.77E-12 |
| GO:017111 | nucleoside-triphosphatase activity | 508/8263 | 1679/36469 | 1.02E-13 | 1.17E-11 |
| GO:016462 | pyrophosphatase activity | 528/8263 | 1757/36469 | 1.17E-13 | 1.29E-11 |
| GO:016817 | hydrolase activity, acting on acid anhydrides | 552/8263 | 1859/36469 | 3.16E-13 | 3.35E-11 |
| GO:043687 | post-translational protein modification | 760/8263 | 2682/36469 | 6.05E-13 | 6.16E-11 |
| GO:016818 | hydrolase activity, acting on acid anhydrides, in phosphorus-containing anhydrides | 535/8263 | 1802/36469 | 7.68E-13 | 7.53E-11 |
| GO:006464 | protein modification process | 836/8263 | 2994/36469 | 1.24E-12 | 1.17E-10 |
| GO:043412 | macromolecule modification | 856/8263 | 3096/36469 | 6.45E-12 | 5.89E-10 |
| GO:008219 | cell death | 81/8263 | 177/36469 | 9.30E-12 | 7.94E-10 |
| GO:016265 | death | 81/8263 | 177/36469 | 9.30E-12 | 7.94E-10 |
| GO:016310 | phosphorylation | 728/8263 | 2591/36469 | 1.17E-11 | 9.68E-10 |
| GO:005529 | sugar binding | 88/8263 | 199/36469 | 1.22E-11 | 9.79E-10 |
| GO:006796 | phosphate metabolic process | 783/8263 | 2826/36469 | 3.69E-11 | 2.79E-09 |
| GO:006793 | phosphorus metabolic process | 783/8263 | 2826/36469 | 3.69E-11 | 2.79E-09 |
| GO:009856 | pollination | 31/8263 | 47/36469 | 2.87E-10 | 1.95E-08 |
| GO:009875 | pollen-pistil interaction | 31/8263 | 47/36469 | 2.87E-10 | 1.95E-08 |
| GO:048544 | recognition of pollen | 31/8263 | 47/36469 | 2.87E-10 | 1.95E-08 |
| GO:048610 | reproductive cellular process | 31/8263 | 47/36469 | 2.87E-10 | 1.95E-08 |
| GO:016787 | hydrolase activity | 1465/8263 | 5681/36469 | 7.56E-10 | 5.00E-08 |
| GO:006259 | DNA metabolic process | 258/8263 | 825/36469 | 5.12E-09 | 3.31E-07 |
| GO:030246 | carbohydrate binding | 113/8263 | 304/36469 | 6.94E-09 | 4.37E-07 |
| GO:007154 | cell communication | 38/8263 | 70/36469 | 9.29E-09 | 5.72E-07 |
| GO:006139 | nucleobase, nucleoside, nucleotide and nucleic acid metabolic process | 727/8263 | 2685/36469 | 1.40E-08 | 8.41E-07 |
| GO:044238 | primary metabolic process | 2939/8263 | 12049/36469 | 1.68E-08 | 9.79E-07 |
| GO:009987 | cellular process | 3216/8263 | 13252/36469 | 1.70E-08 | 9.79E-07 |
|  |  |  |  |  |  |
| GO:016887 | ATPase activity | 239/8263 | 770/36469 | 3.73E-08 | 2.10E-06 |
| GO:006260 | DNA replication | 77/8263 | 194/36469 | 7.38E-08 | 4.07E-06 |
| GO:008037 | cell recognition | 32/8263 | 58/36469 | 8.14E-08 | 4.40E-06 |
| GO:034641 | cellular nitrogen compound metabolic process | 912/8263 | 3476/36469 | 9.92E-08 | 5.25E-06 |
| GO:005875 | microtubule associated complex | 62/8263 | 147/36469 | 1.03E-07 | 5.26E-06 |
| GO:003777 | microtubule motor activity | 62/8263 | 147/36469 | 1.03E-07 | 5.26E-06 |
| GO:003774 | motor activity | 76/8263 | 193/36469 | 1.31E-07 | 6.55E-06 |
| GO:034061 | DNA polymerase activity | 52/8263 | 117/36469 | 1.45E-07 | 7.11E-06 |
| GO:090304 | nucleic acid metabolic process | 524/8263 | 1912/36469 | 3.44E-07 | 1.65E-05 |
| GO:022414 | reproductive process | 36/8263 | 72/36469 | 3.52E-07 | 1.66E-05 |
| GO:006807 | nitrogen compound metabolic process | 939/8263 | 3622/36469 | 5.80E-07 | 2.69E-05 |
| GO:008236 | serine-type peptidase activity | 150/8263 | 462/36469 | 7.31E-07 | 3.28E-05 |
| GO:017171 | serine hydrolase activity | 150/8263 | 462/36469 | 7.31E-07 | 3.28E-05 |
| GO:042623 | ATPase activity, coupled | 180/8263 | 575/36469 | 9.29E-07 | 4.10E-05 |
| GO:043170 | macromolecule metabolic process | 2105/8263 | 8605/36469 | 2.89E-06 | 1.26E-04 |
| GO:003964 | RNA-directed DNA polymerase activity | 33/8263 | 69/36469 | 3.77E-06 | 1.61E-04 |
| GO:050790 | regulation of catalytic activity | 81/8263 | 227/36469 | 5.49E-06 | 2.31E-04 |
| GO:065009 | regulation of molecular function | 81/8263 | 230/36469 | 9.64E-06 | 3.99E-04 |
| GO:044237 | cellular metabolic process | 2576/8263 | 10680/36469 | 1.01E-05 | 4.13E-04 |
| GO:019238 | cyclohydrolase activity | 9/8263 | 10/36469 | 1.25E-05 | 4.94E-04 |
| GO:004252 | serine-type endopeptidase activity | 93/8263 | 274/36469 | 1.25E-05 | 4.94E-04 |
| GO:006278 | RNA-dependent DNA replication | 31/8263 | 67/36469 | 1.70E-05 | 6.63E-04 |
| GO:003674 | molecular_function | 7690/8263 | 33549/36469 | 1.90E-05 | 7.29E-04 |
| GO:003933 | GTP cyclohydrolase activity | 7/8263 | 7/36469 | 3.06E-05 | 1.16E-03 |
| GO:032268 | regulation of cellular protein metabolic process | 15/8263 | 24/36469 | 3.26E-05 | 1.21E-03 |
| GO:006508 | proteolysis | 417/8263 | 1549/36469 | 3.28E-05 | 1.21E-03 |
| GO:050662 | coenzyme binding | 200/8263 | 686/36469 | 3.93E-05 | 1.43E-03 |
| GO:006417 | regulation of translation | 14/8263 | 22/36469 | 4.51E-05 | 1.61E-03 |
| GO:046983 | protein dimerization activity | 210/8263 | 728/36469 | 5.17E-05 | 1.83E-03 |
| GO:044260 | cellular macromolecule metabolic process | 1759/8263 | 7214/36469 | 5.45E-05 | 1.90E-03 |
| GO:008270 | zinc ion binding | 904/8263 | 3578/36469 | 5.63E-05 | 1.94E-03 |
| GO:051248 | negative regulation of protein metabolic process | 13/8263 | 20/36469 | 6.17E-05 | 2.04E-03 |
| GO:017148 | negative regulation of translation | 13/8263 | 20/36469 | 6.17E-05 | 2.04E-03 |
| GO:032269 | negative regulation of cellular protein metabolic process | 13/8263 | 20/36469 | 6.17E-05 | 2.04E-03 |
| GO:070011 | peptidase activity, acting on L-amino acid peptides | 378/8263 | 1404/36469 | 7.48E-05 | 2.44E-03 |
| GO:008152 | metabolic process | 3591/8263 | 15199/36469 | 9.99E-05 | 3.23E-03 |
| GO:010608 | posttranscriptional regulation of gene expression | 15/8263 | 26/36469 | 1.20E-04 | 3.84E-03 |
| GO:008233 | peptidase activity | 383/8263 | 1434/36469 | 1.34E-04 | 4.24E-03 |
| GO:004176 | ATP-dependent peptidase activity | 29/8263 | 67/36469 | 1.40E-04 | 4.35E-03 |
| GO:042802 | identical protein binding | 30/8263 | 71/36469 | 1.82E-04 | 5.62E-03 |
| GO:004518 | nuclease activity | 90/8263 | 281/36469 | 1.85E-04 | 5.62E-03 |
| GO:016763 | transferase activity, transferring pentosyl groups | 19/8263 | 39/36469 | 3.14E-04 | 9.44E-03 |
| GO:004175 | endopeptidase activity | 249/8263 | 906/36469 | 3.32E-04 | 9.87E-03 |
| GO:044281 | small molecule metabolic process | 679/8263 | 2681/36469 | 3.78E-04 | 1.11E-02 |
| GO:048037 | cofactor binding | 259/8263 | 950/36469 | 4.31E-04 | 1.25E-02 |
| GO:009890 | negative regulation of biosynthetic process | 19/8263 | 40/36469 | 4.72E-04 | 1.31E-02 |
| GO:010558 | negative regulation of macromolecule biosynthetic process | 19/8263 | 40/36469 | 4.72E-04 | 1.31E-02 |
| GO:031327 | negative regulation of cellular biosynthetic process | 19/8263 | 40/36469 | 4.72E-04 | 1.31E-02 |
| GO:031324 | negative regulation of cellular metabolic process | 19/8263 | 40/36469 | 4.72E-04 | 1.31E-02 |
| GO:016757 | transferase activity, transferring glycosyl groups | 194/8263 | 694/36469 | 5.82E-04 | 1.57E-02 |
| GO:016875 | ligase activity, forming carbon-oxygen bonds | 63/8263 | 190/36469 | 5.82E-04 | 1.57E-02 |
| GO:016876 | ligase activity, forming aminoacyl-tRNA and related compounds | 63/8263 | 190/36469 | 5.82E-04 | 1.57E-02 |
| GO:003935 | GTP cyclohydrolase II activity | 5/8263 | 5/36469 | 5.97E-04 | 1.60E-02 |
| GO:046128 | purine ribonucleoside metabolic process | 24/8263 | 56/36469 | 6.08E-04 | 1.60E-02 |
| GO:042278 | purine nucleoside metabolic process | 24/8263 | 56/36469 | 6.08E-04 | 1.60E-02 |
| GO:019538 | protein metabolic process | 1606/8263 | 6645/36469 | 6.40E-04 | 1.65E-02 |
| GO:007018 | microtubule-based movement | 74/8263 | 231/36469 | 6.46E-04 | 1.65E-02 |
| GO:004803 | transposase activity | 57/8263 | 169/36469 | 6.47E-04 | 1.65E-02 |
| GO:009892 | negative regulation of metabolic process | 20/8263 | 44/36469 | 6.88E-04 | 1.72E-02 |
| GO:010605 | negative regulation of macromolecule metabolic process | 20/8263 | 44/36469 | 6.88E-04 | 1.72E-02 |
| GO:030597 | RNA glycosylase activity | 10/8263 | 16/36469 | 7.20E-04 | 1.76E-02 |
| GO:030598 | rRNA N-glycosylase activity | 10/8263 | 16/36469 | 7.20E-04 | 1.76E-02 |
| GO:004812 | aminoacyl-tRNA ligase activity | 62/8263 | 188/36469 | 7.49E-04 | 1.82E-02 |
| GO:031072 | heat shock protein binding | 83/8263 | 267/36469 | 8.91E-04 | 2.15E-02 |
| GO:004965 | GABA-B receptor activity | 11/8263 | 19/36469 | 9.59E-04 | 2.29E-02 |
| GO:034660 | ncRNA metabolic process | 103/8263 | 344/36469 | 1.01E-03 | 2.39E-02 |
| GO:006313 | transposition, DNA-mediated | 57/8263 | 172/36469 | 1.05E-03 | 2.40E-02 |
| GO:032196 | transposition | 57/8263 | 172/36469 | 1.05E-03 | 2.40E-02 |
| GO:043039 | tRNA aminoacylation | 60/8263 | 183/36469 | 1.06E-03 | 2.40E-02 |
| GO:043038 | amino acid activation | 60/8263 | 183/36469 | 1.06E-03 | 2.40E-02 |
| GO:000287 | magnesium ion binding | 63/8263 | 194/36469 | 1.06E-03 | 2.40E-02 |
| GO:050660 | FAD binding | 77/8263 | 246/36469 | 1.08E-03 | 2.41E-02 |
| GO:006310 | DNA recombination | 67/8263 | 209/36469 | 1.10E-03 | 2.44E-02 |
| GO:031177 | phosphopantetheine binding | 28/8263 | 71/36469 | 1.10E-03 | 2.44E-02 |
| GO:050661 | NADP or NADPH binding | 38/8263 | 105/36469 | 1.14E-03 | 2.50E-02 |
| GO:006399 | tRNA metabolic process | 85/8263 | 277/36469 | 1.20E-03 | 2.61E-02 |
| GO:006418 | tRNA aminoacylation for protein translation | 59/8263 | 181/36469 | 1.35E-03 | 2.92E-02 |
| GO:043085 | positive regulation of catalytic activity | 14/8263 | 28/36469 | 1.39E-03 | 2.95E-02 |
| GO:044093 | positive regulation of molecular function | 14/8263 | 28/36469 | 1.39E-03 | 2.95E-02 |
| GO:004386 | helicase activity | 88/8263 | 290/36469 | 1.45E-03 | 3.03E-02 |
| GO:043086 | negative regulation of catalytic activity | 24/8263 | 59/36469 | 1.47E-03 | 3.03E-02 |
| GO:016861 | intramolecular oxidoreductase activity, interconverting aldoses and ketoses | 24/8263 | 59/36469 | 1.47E-03 | 3.03E-02 |
| GO:044092 | negative regulation of molecular function | 24/8263 | 59/36469 | 1.47E-03 | 3.03E-02 |
| GO:015630 | microtubule cytoskeleton | 91/8263 | 302/36469 | 1.54E-03 | 3.14E-02 |
| GO:070035 | purine NTP-dependent helicase activity | 57/8263 | 175/36469 | 1.65E-03 | 3.25E-02 |
| GO:008026 | ATP-dependent helicase activity | 57/8263 | 175/36469 | 1.65E-03 | 3.25E-02 |
| GO:016860 | intramolecular oxidoreductase activity | 26/8263 | 66/36469 | 1.66E-03 | 3.25E-02 |
| GO:008483 | transaminase activity | 22/8263 | 53/36469 | 1.68E-03 | 3.25E-02 |
| GO:008417 | fucosyltransferase activity | 11/8263 | 20/36469 | 1.70E-03 | 3.25E-02 |
| GO:016814 | hydrolase activity, acting on carbon-nitrogen (but not peptide) bonds, in cyclic amidines | 11/8263 | 20/36469 | 1.70E-03 | 3.25E-02 |
| GO:008107 | galactoside 2-alpha-L-fucosyltransferase activity | 11/8263 | 20/36469 | 1.70E-03 | 3.25E-02 |
| GO:031127 | alpha(1,2)-fucosyltransferase activity | 11/8263 | 20/36469 | 1.70E-03 | 3.25E-02 |
| GO:016779 | nucleotidyltransferase activity | 119/8263 | 412/36469 | 1.84E-03 | 3.51E-02 |
| GO:048523 | negative regulation of cellular process | 20/8263 | 47/36469 | 1.87E-03 | 3.54E-02 |
| GO:016917 | GABA receptor activity | 13/8263 | 26/36469 | 2.06E-03 | 3.86E-02 |
| GO:051716 | cellular response to stimulus | 75/8263 | 244/36469 | 2.13E-03 | 3.97E-02 |
| GO:044262 | cellular carbohydrate metabolic process | 248/8263 | 931/36469 | 2.17E-03 | 4.01E-02 |
| GO:044430 | cytoskeletal part | 98/8263 | 333/36469 | 2.37E-03 | 4.36E-02 |
| GO:009894 | regulation of catabolic process | 50/8263 | 152/36469 | 2.43E-03 | 4.43E-02 |
| GO:031968 | organelle outer membrane | 10/8263 | 18/36469 | 2.50E-03 | 4.54E-02 |
| GO:033865 | nucleoside bisphosphate metabolic process | 19/8263 | 45/36469 | 2.69E-03 | 4.84E-02 |

**Supplementary Table 5** GO enrichment of genes without SNPs

| GO-ID | Description | Cluster freq | Total freq | P-value | Coor P-value |
| --- | --- | --- | --- | --- | --- |
| GO:048871 | multicellular organismal homeostasis | 433/5853 | 1607/36469 | 4.78E-30 | 1.39E-27 |
| GO:001659 | temperature homeostasis | 433/5853 | 1607/36469 | 4.78E-30 | 1.39E-27 |
| GO:009409 | response to cold | 433/5853 | 1607/36469 | 4.78E-30 | 1.39E-27 |
| GO:050825 | ice binding | 433/5853 | 1607/36469 | 4.78E-30 | 1.39E-27 |
| GO:050824 | water binding | 433/5853 | 1607/36469 | 4.78E-30 | 1.39E-27 |
| GO:050826 | response to freezing | 433/5853 | 1607/36469 | 4.78E-30 | 1.39E-27 |
| GO:042309 | homoiothermy | 433/5853 | 1607/36469 | 4.78E-30 | 1.39E-27 |
| GO:009266 | response to temperature stimulus | 433/5853 | 1610/36469 | 7.39E-30 | 1.85E-27 |
| GO:065008 | regulation of biological quality | 506/5853 | 1966/36469 | 8.15E-30 | 1.85E-27 |
| GO:042592 | homeostatic process | 486/5853 | 1872/36469 | 1.44E-29 | 2.93E-27 |
| GO:009628 | response to abiotic stimulus | 442/5853 | 1683/36469 | 4.69E-28 | 8.68E-26 |
| GO:032501 | multicellular organismal process | 465/5853 | 1806/36469 | 1.88E-27 | 3.19E-25 |
| GO:006950 | response to stress | 576/5853 | 2408/36469 | 2.90E-25 | 4.55E-23 |
| GO:046983 | protein dimerization activity | 225/5853 | 728/36469 | 5.35E-24 | 7.78E-22 |
| GO:050896 | response to stimulus | 615/5853 | 2748/36469 | 1.21E-19 | 1.65E-17 |
| GO:003677 | DNA binding | 939/5853 | 4557/36469 | 1.95E-18 | 2.49E-16 |
| GO:006310 | DNA recombination | 86/5853 | 209/36469 | 3.73E-18 | 4.47E-16 |
| GO:006313 | transposition, DNA-mediated | 75/5853 | 172/36469 | 1.03E-17 | 1.10E-15 |
| GO:032196 | transposition | 75/5853 | 172/36469 | 1.03E-17 | 1.10E-15 |
| GO:004803 | transposase activity | 72/5853 | 169/36469 | 2.09E-16 | 2.13E-14 |
| GO:046914 | transition metal ion binding | 1008/5853 | 5050/36469 | 1.01E-15 | 9.83E-14 |
| GO:005199 | structural constituent of cell wall | 72/5853 | 189/36469 | 2.02E-13 | 1.87E-11 |
| GO:007186 | G-protein coupled receptor protein signaling pathway | 209/5853 | 811/36469 | 5.68E-13 | 5.03E-11 |
| GO:007166 | cell surface receptor linked signaling pathway | 209/5853 | 820/36469 | 1.76E-12 | 1.49E-10 |
| GO:003676 | nucleic acid binding | 1398/5853 | 7490/36469 | 5.25E-12 | 4.28E-10 |
| GO:004872 | receptor activity | 217/5853 | 880/36469 | 1.93E-11 | 1.52E-09 |
| GO:008270 | zinc ion binding | 716/5853 | 3578/36469 | 2.07E-11 | 1.56E-09 |
| GO:065007 | biological regulation | 1091/5853 | 5742/36469 | 4.06E-11 | 2.96E-09 |
| GO:015074 | DNA integration | 32/5853 | 61/36469 | 5.86E-11 | 4.12E-09 |
| GO:005576 | extracellular region | 146/5853 | 558/36469 | 5.65E-10 | 3.84E-08 |
| GO:046872 | metal ion binding | 1138/5853 | 6095/36469 | 1.02E-09 | 6.69E-08 |
| GO:009055 | electron carrier activity | 300/5853 | 1353/36469 | 1.25E-09 | 7.95E-08 |
| GO:043167 | ion binding | 1138/5853 | 6102/36469 | 1.34E-09 | 8.00E-08 |
| GO:043169 | cation binding | 1138/5853 | 6102/36469 | 1.34E-09 | 8.00E-08 |
| GO:004879 | ligand-dependent nuclear receptor activity | 104/5853 | 374/36469 | 5.41E-09 | 3.15E-07 |
| GO:030599 | pectinesterase activity | 50/5853 | 138/36469 | 6.49E-09 | 3.67E-07 |
| GO:022900 | electron transport chain | 48/5853 | 132/36469 | 1.13E-08 | 6.21E-07 |
| GO:004857 | enzyme inhibitor activity | 69/5853 | 223/36469 | 2.16E-08 | 1.16E-06 |
| GO:015035 | protein disulfide oxidoreductase activity | 35/5853 | 88/36469 | 8.11E-08 | 4.24E-06 |
| GO:005507 | copper ion binding | 59/5853 | 186/36469 | 8.32E-08 | 4.24E-06 |
| GO:015036 | disulfide oxidoreductase activity | 35/5853 | 89/36469 | 1.13E-07 | 5.62E-06 |
| GO:004190 | aspartic-type endopeptidase activity | 85/5853 | 309/36469 | 2.13E-07 | 1.01E-05 |
| GO:070001 | aspartic-type peptidase activity | 85/5853 | 309/36469 | 2.13E-07 | 1.01E-05 |
| GO:004930 | G-protein coupled receptor activity | 112/5853 | 443/36469 | 3.61E-07 | 1.67E-05 |
| GO:004888 | transmembrane receptor activity | 119/5853 | 490/36469 | 1.44E-06 | 6.53E-05 |
| GO:051260 | protein homooligomerization | 47/5853 | 150/36469 | 2.40E-06 | 1.04E-04 |
| GO:051259 | protein oligomerization | 47/5853 | 150/36469 | 2.40E-06 | 1.04E-04 |
| GO:009611 | response to wounding | 31/5853 | 85/36469 | 3.89E-06 | 1.65E-04 |
| GO:004867 | serine-type endopeptidase inhibitor activity | 37/5853 | 110/36469 | 4.41E-06 | 1.84E-04 |
| GO:022904 | respiratory electron transport chain | 30/5853 | 82/36469 | 5.14E-06 | 2.09E-04 |
| GO:005488 | binding | 3778/5853 | 22606/36469 | 5.25E-06 | 2.10E-04 |

**Supplementary Table 6** The distribution and annotation of novel SNPs

| Chromosome | Sum | Intergenic | Genic | CDS | | | | | | 5’UTR | | 3’UTR | | Intron | |  |
| --- | --- | --- | --- | --- | --- | --- | --- | --- | --- | --- | --- | --- | --- | --- | --- | --- |
|  |  |  |  | Total | | Syn | | Nonsyn | |  |  |  |  |  |  |  |
| chr1 | 44,904 | 28,110 | 16,794 | | 5,343 | | 1,952 | | 3,391 | | 1,787 | | 2,004 | | 7,660 | |
| chr2 | 38,011 | 23,542 | 14,469 | | 4,718 | | 1,727 | | 2,991 | | 1,543 | | 1,921 | | 6,287 | |
| chr3 | 31,662 | 19,935 | 11,727 | | 3,791 | | 1,381 | | 2,410 | | 1,293 | | 1,432 | | 5,211 | |
| chr4 | 36,825 | 24,397 | 12,428 | | 4,014 | | 1,481 | | 2,533 | | 1,267 | | 1,601 | | 5,546 | |
| chr5 | 32,037 | 19,761 | 12,276 | | 4,001 | | 1,475 | | 2,526 | | 1,152 | | 1,652 | | 5,471 | |
| chr6 | 25,921 | 15,992 | 9,929 | | 3,183 | | 1,178 | | 2,005 | | 1,093 | | 1,463 | | 4,190 | |
| chr7 | 26,648 | 17,321 | 9,327 | | 3,074 | | 1,189 | | 1,885 | | 993 | | 1,215 | | 4,045 | |
| chr8 | 27,534 | 17,345 | 10,189 | | 3,277 | | 1,117 | | 2,160 | | 949 | | 1,503 | | 4,460 | |
| chr9 | 24,299 | 15,445 | 8,854 | | 2,754 | | 990 | | 1,764 | | 868 | | 1,139 | | 4,093 | |
| chr10 | 22,549 | 14,117 | 8,432 | | 2,893 | | 1,050 | | 1,843 | | 836 | | 1,001 | | 3,702 | |
| chr0 | 2,121 | 1,596 | 525 | | 150 | | 68 | | 82 | | 29 | | 54 | | 292 | |
| total | 312,511 | 197,561 | 114,950 | | 37,198 | | 13,608 | | 23,590 | | 11,810 | | 14,985 | | 50,957 | |

**Supplementary Table 7 The ten genes most enriched in novel SNPs.**

| Rank | Gene ID | CDS | Intron | 5’UTR | 3’UTR | Sum | Function annotated by InterPro |
| --- | --- | --- | --- | --- | --- | --- | --- |
| 1 | GRMZM2G159715_P01 | 24 | 88 | 1 | 1 | 114 | Transcription factor TFIIB related |
| 2 | GRMZM2G452960_P01 | 3 | 0 | 5 | 103 | 111 | Unknown |
| 3 | GRMZM2G113750_P01 | 9 | 91 | 3 | 0 | 103 | Tubulin/FtsZ, GTPase |
| 4 | GRMZM2G456367_P01 | 6 | 86 | 0 | 0 | 92 | Armadillo-type fold |
| 5 | GRMZM2G384863_P01 | 13 | 75 | 0 | 0 | 88 | High mobility group, superfamily |
| 6 | GRMZM2G087944_P01 | 19 | 61 | 0 | 0 | 80 | Pathogenesis-related transcriptional factor and ERF, DNA-binding |
| 7 | GRMZM2G056996_P01 | 0 | 0 | 77 | 0 | 77 | Pentatricopeptide repeat |
| 8 | GRMZM2G392176_P01 | 6 | 68 | 0 | 3 | 77 | Tyrosine protein kinase |
| 9 | GRMZM2G180254_P01 | 36 | 0 | 0 | 38 | 74 | ATPase, AAA+ type, core |
| 10 | GRMZM2G305211_P01 | 16 | 56 | 0 | 0 | 72 | 14-3-3 protein |

**Supplementary Table 8** GO enrichment of genes with novel large effect SNPs

| GO-ID | Description | Cluster freq | Total freq | P-value | Coor P-value |
| --- | --- | --- | --- | --- | --- |
| GO:000166 | nucleotide binding | 665/3112 | 3751/23688 | 1.17E-18 | 2.39E-15 |
| GO:032555 | purine ribonucleotide binding | 606/3112 | 3383/23688 | 7.07E-18 | 4.82E-15 |
| GO:032553 | ribonucleotide binding | 606/3112 | 3383/23688 | 7.07E-18 | 4.82E-15 |
| GO:017076 | purine nucleotide binding | 630/3112 | 3561/23688 | 2.17E-17 | 1.11E-14 |
| GO:005524 | ATP binding | 549/3112 | 3034/23688 | 5.28E-17 | 2.16E-14 |
| GO:032559 | adenyl ribonucleotide binding | 549/3112 | 3038/23688 | 6.92E-17 | 2.36E-14 |
| GO:030554 | adenyl nucleotide binding | 571/3112 | 3209/23688 | 3.50E-16 | 8.48E-14 |
| GO:001883 | purine nucleoside binding | 571/3112 | 3209/23688 | 3.50E-16 | 8.48E-14 |
| GO:001882 | nucleoside binding | 571/3112 | 3210/23688 | 3.73E-16 | 8.48E-14 |
| GO:003824 | catalytic activity | 1577/3112 | 10639/23688 | 2.63E-12 | 5.39E-10 |
| GO:009987 | cellular process | 1253/3112 | 8312/23688 | 7.02E-11 | 1.31E-08 |
| GO:044238 | primary metabolic process | 1158/3112 | 7616/23688 | 7.91E-11 | 1.35E-08 |
| GO:017111 | nucleoside-triphosphatase activity | 214/3112 | 1116/23688 | 3.97E-09 | 6.25E-07 |
| GO:016817 | hydrolase activity, acting on acid anhydrides | 230/3112 | 1224/23688 | 6.36E-09 | 9.29E-07 |
| GO:008152 | metabolic process | 1394/3112 | 9507/23688 | 8.25E-09 | 1.12E-06 |
| GO:016462 | pyrophosphatase activity | 220/3112 | 1167/23688 | 1.05E-08 | 1.34E-06 |
| GO:044237 | cellular metabolic process | 1007/3112 | 6658/23688 | 1.24E-08 | 1.46E-06 |
| GO:016818 | hydrolase activity, acting on acid anhydrides, in phosphorus-containing anhydrides | 223/3112 | 1189/23688 | 1.29E-08 | 1.46E-06 |
| GO:043170 | macromolecule metabolic process | 835/3112 | 5485/23688 | 1.51E-07 | 1.63E-05 |
| GO:019538 | protein metabolic process | 653/3112 | 4181/23688 | 1.60E-07 | 1.63E-05 |
| GO:044106 | cellular amine metabolic process | 106/3112 | 508/23688 | 6.88E-07 | 6.69E-05 |
| GO:043234 | protein complex | 218/3112 | 1217/23688 | 7.20E-07 | 6.69E-05 |
| GO:044260 | cellular macromolecule metabolic process | 693/3112 | 4517/23688 | 9.05E-07 | 8.04E-05 |
| GO:016787 | hydrolase activity | 576/3112 | 3708/23688 | 2.20E-06 | 1.87E-04 |
| GO:044267 | cellular protein metabolic process | 513/3112 | 3269/23688 | 2.84E-06 | 2.33E-04 |
| GO:006520 | cellular amino acid metabolic process | 97/3112 | 471/23688 | 3.63E-06 | 2.80E-04 |
| GO:016772 | transferase activity, transferring phosphorus-containing groups | 355/3112 | 2173/23688 | 3.77E-06 | 2.80E-04 |
| GO:009308 | amine metabolic process | 115/3112 | 582/23688 | 3.88E-06 | 2.80E-04 |
| GO:019318 | hexose metabolic process | 63/3112 | 272/23688 | 3.99E-06 | 2.80E-04 |
| GO:005996 | monosaccharide metabolic process | 67/3112 | 295/23688 | 4.10E-06 | 2.80E-04 |
| GO:016773 | phosphotransferase activity, alcohol group as acceptor | 291/3112 | 1742/23688 | 5.23E-06 | 3.40E-04 |
| GO:009056 | catabolic process | 111/3112 | 561/23688 | 5.33E-06 | 3.40E-04 |
| GO:016740 | transferase activity | 533/3112 | 3444/23688 | 9.00E-06 | 5.58E-04 |
| GO:016070 | RNA metabolic process | 104/3112 | 525/23688 | 9.88E-06 | 5.94E-04 |
| GO:006006 | glucose metabolic process | 58/3112 | 251/23688 | 1.02E-05 | 5.95E-04 |
| GO:004672 | protein kinase activity | 251/3112 | 1486/23688 | 1.08E-05 | 6.16E-04 |
| GO:006468 | protein amino acid phosphorylation | 247/3112 | 1460/23688 | 1.14E-05 | 6.30E-04 |
| GO:034641 | cellular nitrogen compound metabolic process | 359/3112 | 2232/23688 | 1.37E-05 | 7.40E-04 |
| GO:043687 | post-translational protein modification | 288/3112 | 1746/23688 | 1.60E-05 | 8.40E-04 |
| GO:016301 | kinase activity | 297/3112 | 1816/23688 | 2.30E-05 | 1.17E-03 |
| GO:006807 | nitrogen compound metabolic process | 371/3112 | 2330/23688 | 2.41E-05 | 1.20E-03 |
| GO:034660 | ncRNA metabolic process | 50/3112 | 213/23688 | 2.59E-05 | 1.26E-03 |
| GO:044281 | small molecule metabolic process | 275/3112 | 1669/23688 | 2.72E-05 | 1.29E-03 |
| GO:044275 | cellular carbohydrate catabolic process | 45/3112 | 187/23688 | 3.36E-05 | 1.49E-03 |
| GO:046164 | alcohol catabolic process | 45/3112 | 187/23688 | 3.36E-05 | 1.49E-03 |
| GO:042623 | ATPase activity, coupled | 81/3112 | 398/23688 | 3.44E-05 | 1.49E-03 |
| GO:006007 | glucose catabolic process | 44/3112 | 182/23688 | 3.63E-05 | 1.49E-03 |
| GO:046365 | monosaccharide catabolic process | 44/3112 | 182/23688 | 3.63E-05 | 1.49E-03 |
| GO:019320 | hexose catabolic process | 44/3112 | 182/23688 | 3.63E-05 | 1.49E-03 |
| GO:043412 | macromolecule modification | 318/3112 | 1972/23688 | 3.63E-05 | 1.49E-03 |
| GO:006464 | protein modification process | 310/3112 | 1919/23688 | 4.00E-05 | 1.60E-03 |
| GO:044248 | cellular catabolic process | 54/3112 | 240/23688 | 4.41E-05 | 1.73E-03 |
| GO:016887 | ATPase activity | 102/3112 | 532/23688 | 4.77E-05 | 1.84E-03 |
| GO:044282 | small molecule catabolic process | 54/3112 | 241/23688 | 4.96E-05 | 1.88E-03 |
| GO:006519 | cellular amino acid and derivative metabolic process | 107/3112 | 565/23688 | 5.31E-05 | 1.98E-03 |
| GO:043436 | oxoacid metabolic process | 127/3112 | 697/23688 | 6.97E-05 | 2.50E-03 |
| GO:019752 | carboxylic acid metabolic process | 127/3112 | 697/23688 | 6.97E-05 | 2.50E-03 |
| GO:004812 | aminoacyl-tRNA ligase activity | 33/3112 | 127/23688 | 7.44E-05 | 2.58E-03 |
| GO:006082 | organic acid metabolic process | 127/3112 | 698/23688 | 7.44E-05 | 2.58E-03 |
| GO:006418 | tRNA aminoacylation for protein translation | 32/3112 | 122/23688 | 7.81E-05 | 2.66E-03 |
| GO:016875 | ligase activity, forming carbon-oxygen bonds | 33/3112 | 128/23688 | 8.77E-05 | 2.89E-03 |
| GO:016876 | ligase activity, forming aminoacyl-tRNA and related compounds | 33/3112 | 128/23688 | 8.77E-05 | 2.89E-03 |
| GO:044262 | cellular carbohydrate metabolic process | 106/3112 | 566/23688 | 9.08E-05 | 2.91E-03 |
| GO:043039 | tRNA aminoacylation | 32/3112 | 123/23688 | 9.25E-05 | 2.91E-03 |
| GO:043038 | amino acid activation | 32/3112 | 123/23688 | 9.25E-05 | 2.91E-03 |
| GO:004674 | protein serine/threonine kinase activity | 227/3112 | 1371/23688 | 1.03E-04 | 3.19E-03 |
| GO:042180 | cellular ketone metabolic process | 127/3112 | 704/23688 | 1.09E-04 | 3.33E-03 |
| GO:007018 | microtubule-based movement | 34/3112 | 135/23688 | 1.14E-04 | 3.42E-03 |
| GO:006399 | tRNA metabolic process | 41/3112 | 176/23688 | 1.57E-04 | 4.54E-03 |
| GO:007017 | microtubule-based process | 41/3112 | 176/23688 | 1.57E-04 | 4.54E-03 |
| GO:012501 | programmed cell death | 31/3112 | 121/23688 | 1.60E-04 | 4.54E-03 |
| GO:006915 | apoptosis | 31/3112 | 121/23688 | 1.60E-04 | 4.54E-03 |
| GO:044265 | cellular macromolecule catabolic process | 36/3112 | 150/23688 | 2.07E-04 | 5.79E-03 |
| GO:015630 | microtubule cytoskeleton | 42/3112 | 184/23688 | 2.11E-04 | 5.84E-03 |
| GO:008219 | cell death | 34/3112 | 140/23688 | 2.41E-04 | 6.48E-03 |
| GO:016265 | death | 34/3112 | 140/23688 | 2.41E-04 | 6.48E-03 |
| GO:043632 | modification-dependent macromolecule catabolic process | 35/3112 | 147/23688 | 2.96E-04 | 7.39E-03 |
| GO:051603 | proteolysis involved in cellular protein catabolic process | 35/3112 | 147/23688 | 2.96E-04 | 7.39E-03 |
| GO:044257 | cellular protein catabolic process | 35/3112 | 147/23688 | 2.96E-04 | 7.39E-03 |
| GO:006511 | ubiquitin-dependent protein catabolic process | 35/3112 | 147/23688 | 2.96E-04 | 7.39E-03 |
| GO:019941 | modification-dependent protein catabolic process | 35/3112 | 147/23688 | 2.96E-04 | 7.39E-03 |
| GO:004386 | helicase activity | 41/3112 | 181/23688 | 2.96E-04 | 7.39E-03 |
| GO:006096 | glycolysis | 36/3112 | 153/23688 | 3.12E-04 | 7.69E-03 |
| GO:044445 | cytosolic part | 24/3112 | 89/23688 | 3.75E-04 | 9.02E-03 |
| GO:005829 | cytosol | 24/3112 | 89/23688 | 3.75E-04 | 9.02E-03 |
| GO:044430 | cytoskeletal part | 44/3112 | 205/23688 | 6.26E-04 | 1.49E-02 |
| GO:016052 | carbohydrate catabolic process | 50/3112 | 241/23688 | 6.37E-04 | 1.50E-02 |
| GO:031072 | heat shock protein binding | 40/3112 | 183/23688 | 7.41E-04 | 1.72E-02 |
| GO:006508 | proteolysis | 173/3112 | 1049/23688 | 8.06E-04 | 1.85E-02 |
| GO:005875 | microtubule associated complex | 25/3112 | 99/23688 | 8.29E-04 | 1.86E-02 |
| GO:003777 | microtubule motor activity | 25/3112 | 99/23688 | 8.29E-04 | 1.86E-02 |
| GO:006066 | alcohol metabolic process | 78/3112 | 422/23688 | 1.06E-03 | 2.36E-02 |
| GO:016861 | intramolecular oxidoreductase activity, interconverting aldoses and ketoses | 13/3112 | 40/23688 | 1.28E-03 | 2.80E-02 |
| GO:070035 | purine NTP-dependent helicase activity | 27/3112 | 113/23688 | 1.30E-03 | 2.80E-02 |
| GO:008026 | ATP-dependent helicase activity | 27/3112 | 113/23688 | 1.30E-03 | 2.80E-02 |
| GO:016639 | oxidoreductase activity, acting on the CH-NH2 group of donors, NAD or NADP as acceptor | 4/3112 | 5/23688 | 1.33E-03 | 2.83E-02 |
| GO:016860 | intramolecular oxidoreductase activity | 14/3112 | 45/23688 | 1.37E-03 | 2.88E-02 |
| GO:032991 | macromolecular complex | 326/3112 | 2133/23688 | 1.39E-03 | 2.89E-02 |
| GO:005488 | binding | 2051/3112 | 15045/23688 | 1.51E-03 | 2.89E-02 |
| GO:043087 | regulation of GTPase activity | 23/3112 | 92/23688 | 1.52E-03 | 2.89E-02 |
| GO:051336 | regulation of hydrolase activity | 23/3112 | 92/23688 | 1.52E-03 | 2.89E-02 |
| GO:006140 | regulation of nucleotide metabolic process | 23/3112 | 92/23688 | 1.52E-03 | 2.89E-02 |
| GO:033124 | regulation of GTP catabolic process | 23/3112 | 92/23688 | 1.52E-03 | 2.89E-02 |
| GO:033121 | regulation of purine nucleotide catabolic process | 23/3112 | 92/23688 | 1.52E-03 | 2.89E-02 |
| GO:031329 | regulation of cellular catabolic process | 23/3112 | 92/23688 | 1.52E-03 | 2.89E-02 |
| GO:030811 | regulation of nucleotide catabolic process | 23/3112 | 92/23688 | 1.52E-03 | 2.89E-02 |
| GO:005975 | carbohydrate metabolic process | 172/3112 | 1057/23688 | 1.52E-03 | 2.89E-02 |
| GO:005839 | proteasome core complex | 12/3112 | 36/23688 | 1.53E-03 | 2.89E-02 |
| GO:006139 | nucleobase, nucleoside, nucleotide and nucleic acid metabolic process | 276/3112 | 1784/23688 | 1.63E-03 | 3.06E-02 |
| GO:044428 | nuclear part | 35/3112 | 161/23688 | 1.69E-03 | 3.14E-02 |
| GO:016310 | phosphorylation | 266/3112 | 1715/23688 | 1.72E-03 | 3.16E-02 |
| GO:009894 | regulation of catabolic process | 24/3112 | 99/23688 | 1.90E-03 | 3.48E-02 |
| GO:016874 | ligase activity | 78/3112 | 432/23688 | 2.07E-03 | 3.75E-02 |
| GO:004518 | nuclease activity | 40/3112 | 193/23688 | 2.13E-03 | 3.80E-02 |
| GO:051236 | establishment of RNA localization | 3/3112 | 3/23688 | 2.27E-03 | 3.80E-02 |
| GO:016886 | ligase activity, forming phosphoric ester bonds | 3/3112 | 3/23688 | 2.27E-03 | 3.80E-02 |
| GO:050657 | nucleic acid transport | 3/3112 | 3/23688 | 2.27E-03 | 3.80E-02 |
| GO:050658 | RNA transport | 3/3112 | 3/23688 | 2.27E-03 | 3.80E-02 |
| GO:006403 | RNA localization | 3/3112 | 3/23688 | 2.27E-03 | 3.80E-02 |
| GO:045181 | glutamate synthase activity, NADH or NADPH as acceptor | 3/3112 | 3/23688 | 2.27E-03 | 3.80E-02 |
| GO:003910 | DNA ligase (ATP) activity | 3/3112 | 3/23688 | 2.27E-03 | 3.80E-02 |
| GO:003909 | DNA ligase activity | 3/3112 | 3/23688 | 2.27E-03 | 3.80E-02 |
| GO:005515 | protein binding | 446/3112 | 3017/23688 | 2.55E-03 | 4.25E-02 |
| GO:000502 | proteasome complex | 13/3112 | 43/23688 | 2.67E-03 | 4.35E-02 |
| GO:005945 | 6-phosphofructokinase complex | 15/3112 | 53/23688 | 2.68E-03 | 4.35E-02 |
| GO:003872 | 6-phosphofructokinase activity | 15/3112 | 53/23688 | 2.68E-03 | 4.35E-02 |
| GO:004713 | protein tyrosine kinase activity | 200/3112 | 1267/23688 | 2.84E-03 | 4.58E-02 |
| GO:051258 | protein polymerization | 20/3112 | 80/23688 | 2.98E-03 | 4.76E-02 |
| GO:006796 | phosphate metabolic process | 283/3112 | 1855/23688 | 3.15E-03 | 4.96E-02 |
| GO:006793 | phosphorus metabolic process | 283/3112 | 1855/23688 | 3.15E-03 | 4.96E-02 |

**Supplementary Table 9** Four novel genes in Four-row Wax

| Homologs | CDS_Length of novel gene (bp) | Predicted function |
| --- | --- | --- |
| Os03t0411800-01 | 933 | zinc transporter 2 |
| Sb01g003150.1 | 1803 | Pentatricopeptide repeat-containing protein |
| Sb04g036860.1 | 783 | Phloem-specific lectin |
| Sb03g004770.1 | 690 | Saposin-like type B |

****Supplementary Table 10 Isolated 4 genes contributing to changing KRN.****

| Gene | Homology(function) | ID | Chr | Ref |
| --- | --- | --- | --- | --- |
| knotted1(kn1) | Homeobox | GRMZM2G017087 | 1 | Vollbrecht et al.,1991 |
| terminalear1(te1) | RNA-binding | GRMZM2G085113 | 3 | Veit et al., 1998 |
| fasciated ear2(fea2) | LRR receptor-like protein | GRMZM2G104925 | 4 | Bommert et al., 2013 |
| thick tasseldwarf1(td1) | LRR receptor-like kinase | GRMZM2G300133 | 5 | Bommert et al., 2005 |

****Supplementary Table 11 Variation in genes contributing to changing KRN, in Four-row Wax.****

| Gene | ID | Indel | SNP | 5k upstream of gene |
| --- | --- | --- | --- | --- |
| knotted1(kn1) | GRMZM2G017087 | 2(intron) | 1(CDS) | 1 SV, 9 SNP |
| terminalear1(te1) | GRMZM2G085113 | 5(1(CDS),1(3’UTR),3(intron)) | 2(1(intron),1(CDS)) | 3 Indel, 4 SNP |
| fasciated ear2(fea2) | GRMZM2G104925 | 0 | 6(3(3’UTR),3(CDS)) | 7 Indel, 1 SV, 55 SNP |
| thick tasseldwarf1(td1) | GRMZM2G300133 | 0 | 2(1(3’UTR),1(CDS)) | 2 Indel, 1 SV, 22 SNP |

**Supplementary Table 12 Five large effect QTLs contributing to changing KRN.**

| Background | Number and type of genetic material | Flankingmarkers | LOD | R^2^(%) | Chromo-some | Start position | End position | Reference |
| --- | --- | --- | --- | --- | --- | --- | --- | --- |
| Y478 x D340 | 397 F2:3 | phi127–mmc0191 | 15.1 | 13.05 | 2 | 186767370 | 196464832 | Liu et al.[2010] |
| Y478 x D340 | 397 F2:3 | bnlg339–umc1865 | 22.2 | 17.86 | 7 | 131317856 | 141592310 | Liu et al.[2010] |
| Zong3 x 871 | 294 F8 RILs | umc1460-umc1562 | 22.9 | 8 | 8 | 109480749 | 124519245 | Ma et al.[2007] |
| Dan232 x N04 | 220 BC2F2 | umc2017-umc1877 | 3.0 | 9 | 10 | 62062979 | 145731873 | Li et al.[2007] |
| Zong3 x 871 | 266 F2:3 | bnlg127-csu147 | 6.6 | 13.1 | 9 | 49716451 | 97782157 | Yan et al.[2006] |

**Supplementary Table 13 Variations in five QTL regions in Four-row Wax.**

| Region of QTL | DNA polymorphisms | Sum | Density(/Mb) | Intergenic | Genic | CDS | 5'UTR | 3'UTR | Intron |
| --- | --- | --- | --- | --- | --- | --- | --- | --- | --- |
| phi127_mmc0191 | SNPs | 6119 | 631 | 4072 | 2047 | 598 | 160 | 227 | 1062 |
|  | Novel SNPs | 233 | 24 | 190 | 43 | 8 | 5 | 6 | 24 |
|  | InDel | 1019 | 105 | 677 | 342 | 31 | 35 | 56 | 220 |
|  | SV | 180 | 19 | 101 | 79 | 39 | 13 | 16 | 11 |
| bnlg339_umc1865 | SNPs | 8914 | 868 | 6572 | 2342 | 614 | 201 | 312 | 1215 |
|  | Novel SNPs | 323 | 31 | 264 | 59 | 11 | 3 | 7 | 38 |
|  | InDel | 1301 | 127 | 781 | 520 | 31 | 49 | 82 | 358 |
|  | SV | 205 | 20 | 127 | 78 | 29 | 12 | 19 | 18 |
| umc1460_umc1562 | SNPs | 16151 | 1074 | 12694 | 3457 | 1003 | 349 | 428 | 1677 |
|  | Novel SNPs | 496 | 33 | 388 | 108 | 11 | 9 | 18 | 70 |
|  | InDel | 1493 | 99 | 1031 | 462 | 31 | 49 | 72 | 310 |
|  | SV | 240 | 16 | 168 | 72 | 26 | 12 | 15 | 19 |
| bnlg127_csu147 | SNPs | 40316 | 839 | 35199 | 5117 | 1426 | 579 | 524 | 2588 |
|  | Novel SNPs | 593 | 12 | 531 | 62 | 7 | 6 | 9 | 40 |
|  | InDel | 3911 | 81 | 3230 | 681 | 45 | 71 | 94 | 471 |
|  | SV | 782 | 16 | 580 | 202 | 102 | 37 | 33 | 30 |
| umc2017_umc1877 | SNPs | 86769 | 1037 | 69515 | 17254 | 5261 | 1896 | 1980 | 8117 |
|  | Novel SNPs | 1668 | 20 | 1408 | 260 | 35 | 27 | 43 | 155 |
|  | InDel | 8669 | 104 | 6090 | 2579 | 203 | 311 | 395 | 1670 |
|  | SV | 1507 | 18 | 1008 | 499 | 249 | 73 | 84 | 93 |

**Supplementary Table 14 Potential causal genes of the reduced kernel row trait in Four-row Wax.**

| Chromosome | Gene ID | CDS length |
| --- | --- | --- |
| chr10 | GRMZM2G062807_P01 | 523 |
| chr10 | GRMZM2G111482_P05 | 825 |
| chr10 | GRMZM2G111462_P01 | 3912 |
| chr10 | GRMZM2G030009_P02 | 1582 |
| chr10 | GRMZM2G497555_P01 | 343 |
| chr10 | GRMZM2G030159_P01 | 225 |
| chr10 | GRMZM2G096107_P01 | 4619 |
| chr10 | GRMZM2G096135_P01 | 2241 |
| chr10 | GRMZM2G048656_P01 | 339 |
| chr10 | GRMZM2G392176_P01 | 4313 |
| chr10 | GRMZM2G410191_P01 | 513 |
| chr10 | GRMZM2G111423_P01 | 1236 |
| chr10 | AC183938.4_FGP011 | 825 |
| chr10 | GRMZM2G396846_P01 | 2019 |
| chr2 | GRMZM2G164008_P01 | 1093 |
| chr2 | GRMZM2G025933_P01 | 465 |
| chr2 | GRMZM2G336239_P01 | 615 |
| chr2 | GRMZM2G153369_P02 | 682 |
| chr2 | GRMZM2G153523_P01 | 2165 |
| chr2 | GRMZM2G453753_P01 | 1976 |
| chr2 | GRMZM2G153575_P01 | 1626 |
| chr2 | GRMZM2G164005_P01 | 544 |
| chr2 | GRMZM2G153586_P01 | 1579 |
| chr2 | GRMZM2G164426_P01 | 1365 |
| chr2 | GRMZM2G164450_P01 | 483 |
| chr2 | GRMZM2G466243_P01 | 3338 |
| chr2 | GRMZM2G105885_P01 | 228 |
| chr2 | GRMZM2G105869_P02 | 1458 |
| chr7 | GRMZM2G000290_P01 | 1731 |
| chr7 | GRMZM2G482720_P01 | 888 |
| chr7 | GRMZM2G000264_P01 | 3836 |
| chr7 | GRMZM2G548616_P01 | 565 |
| chr7 | GRMZM2G110500_P01 | 1690 |
| chr7 | GRMZM2G505873_P01 | 616 |
| chr7 | AC193647.3_FGP005 | 621 |
| chr7 | GRMZM2G468044_P01 | 153 |
| chr7 | GRMZM2G102243_P01 | 3554 |
| chr7 | GRMZM2G000281_P01 | 1666 |
| chr7 | GRMZM2G410595_P01 | 870 |
| chr7 | GRMZM2G041462_P01 | 1641 |
| chr7 | GRMZM2G338916_P01 | 649 |
| chr7 | GRMZM2G338928_P01 | 3180 |
| chr7 | GRMZM2G041664_P01 | 393 |
| chr7 | GRMZM2G041666_P01 | 1579 |
| chr7 | GRMZM2G080507_P01 | 2409 |
| chr7 | GRMZM2G080543_P01 | 290 |
| chr7 | GRMZM2G468047_P01 | 902 |
| chr8 | AC194109.3_FGP005 | 426 |
| chr8 | GRMZM2G010319_P01 | 744 |
| chr8 | GRMZM2G062550_P01 | 605 |
| chr8 | GRMZM2G497742_P01 | 265 |
| chr8 | GRMZM2G325296_P02 | 1277 |
| chr8 | GRMZM2G104283_P01 | 1918 |
| chr8 | GRMZM2G130529_P01 | 627 |
| chr8 | GRMZM2G048713_P01 | 567 |
| chr8 | GRMZM2G350023_P01 | 1494 |
| chr8 | GRMZM2G136369_P01 | 1816 |
| chr8 | GRMZM2G122736_P01 | 738 |
| chr8 | GRMZM2G122720_P01 | 344 |
| chr8 | AC233864.1_FGP004 | 1467 |
| chr8 | AC210792.5_FGP003 | 429 |
| chr8 | GRMZM2G456454_P01 | 2043 |
| chr8 | GRMZM2G132696_P01 | 518 |
| chr8 | GRMZM2G010442_P01 | 666 |
| chr8 | GRMZM2G010452_P01 | 5613 |
| chr8 | GRMZM2G062555_P01 | 2185 |
| chr8 | AC201832.3_FGP006 | 576 |
| chr8 | GRMZM2G497745_P01 | 324 |
| chr8 | GRMZM2G034815_P01 | 1545 |
| chr8 | GRMZM2G034647_P01 | 1796 |
| chr8 | GRMZM2G130534_P01 | 498 |
| chr8 | GRMZM2G367072_P01 | 1728 |
| chr8 | GRMZM2G309578_P01 | 1467 |
| chr8 | GRMZM2G350006_P01 | 472 |
| chr9 | GRMZM2G059229_P01 | 555 |
| chr9 | GRMZM2G059233_P01 | 460 |
| chr9 | AC198122.3_FGP002 | 957 |
| chr9 | GRMZM2G110700_P01 | 392 |
| chr9 | GRMZM2G087635_P01 | 2359 |
| chr9 | GRMZM2G387307_P01 | 1786 |
| chr9 | GRMZM2G052923_P01 | 544 |
| chr9 | GRMZM2G416875_P01 | 1325 |
| chr9 | GRMZM2G357036_P01 | 602 |
| chr9 | GRMZM2G387196_P01 | 1999 |
| chr9 | GRMZM2G087712_P01 | 3613 |
| chr9 | GRMZM2G087806_P01 | 2800 |
| chr9 | GRMZM2G116056_P01 | 713 |
| chr9 | GRMZM2G416886_P01 | 718 |
