# Supplementary material for: Characterization of Genome-Wide Variation in Four-Row Wax, a Waxy Maize Landrace with a Reduced Kernel Row Phenotype
Source: Front Plant Sci. 2016 May 18;7:667. doi: 10.3389/fpls.2016.00667 (PMC4870249; doi:10.3389/fpls.2016.00667)

*Supplementary Material*

**Characterization of genome-wide variations in Four-row Wax, a waxy maize landrace with a reduced kernel row phenotype**

**Hanmei Liu^1†^,Xuewen Wang^5†^, Bin Wei^2†^, Yongbin Wang^2^, Yinghong Liu^2^, Junjie Zhang^1^, Yufeng Hu^3^, Guowu Yu^3^, Jian Li^4^, Zhanbin Xu^4^, Yubi Huang^2,3,^***

***Correspondence:**Yubi Huang, [yubihuang@sohu.com](mailto:yubihuang@sohu.com)

Additional file 1:Supplementary Figures S1 to S6

Supplementary FigureLegends

**Figure S1 GO enrichment of genes with large effect SNPs.**

**Figure S2** The distribution of number and distance between adjacent SNPs.

**Figure S3 GO enrichment of genes without SNPs.**

**Figure S4 GO enrichment of genes with novel large effect SNPs.**

**Figure S5** A phylogenetic tree for 7 re-sequenced maize lines.

Figure S6 SNPs densities in maize genomic regions associated with kernel row number (on chromosomes 2, 8, 9 and 10).

Total of 1,747,402 SNPs (collections ofSNPs identified in 6 elite inbred lines) in these maize lines were used to reconstruct the phylogenetic tree.


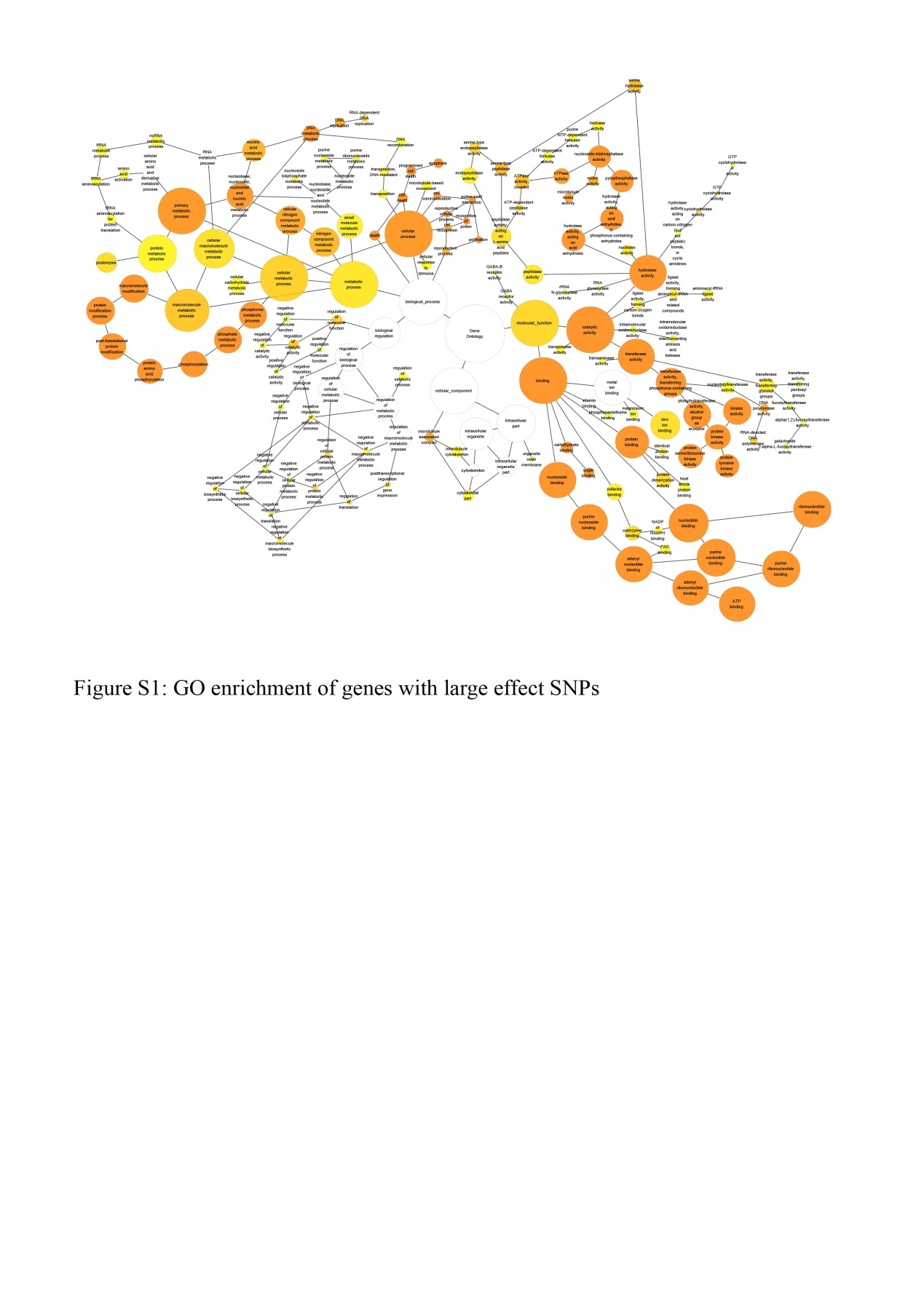

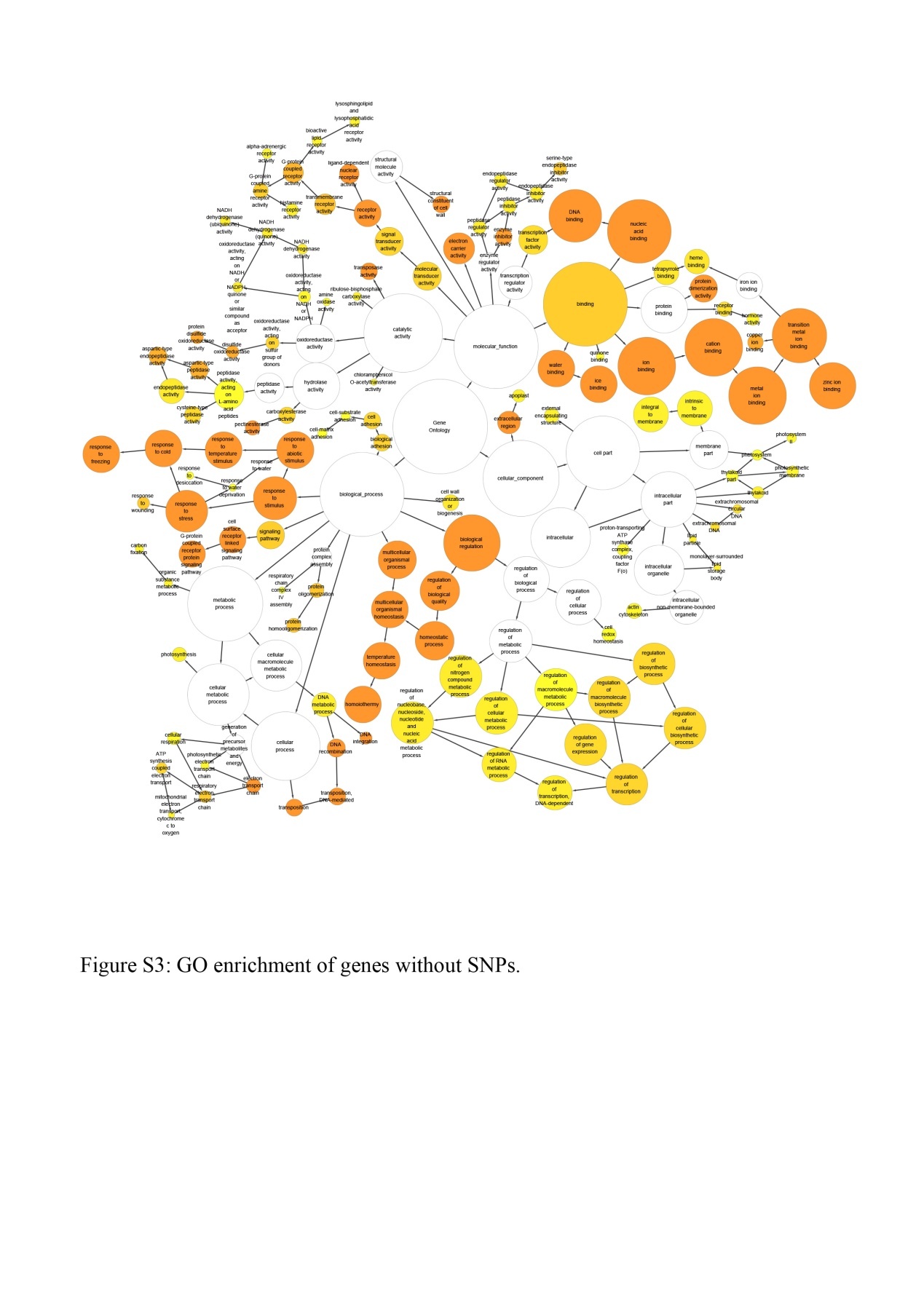


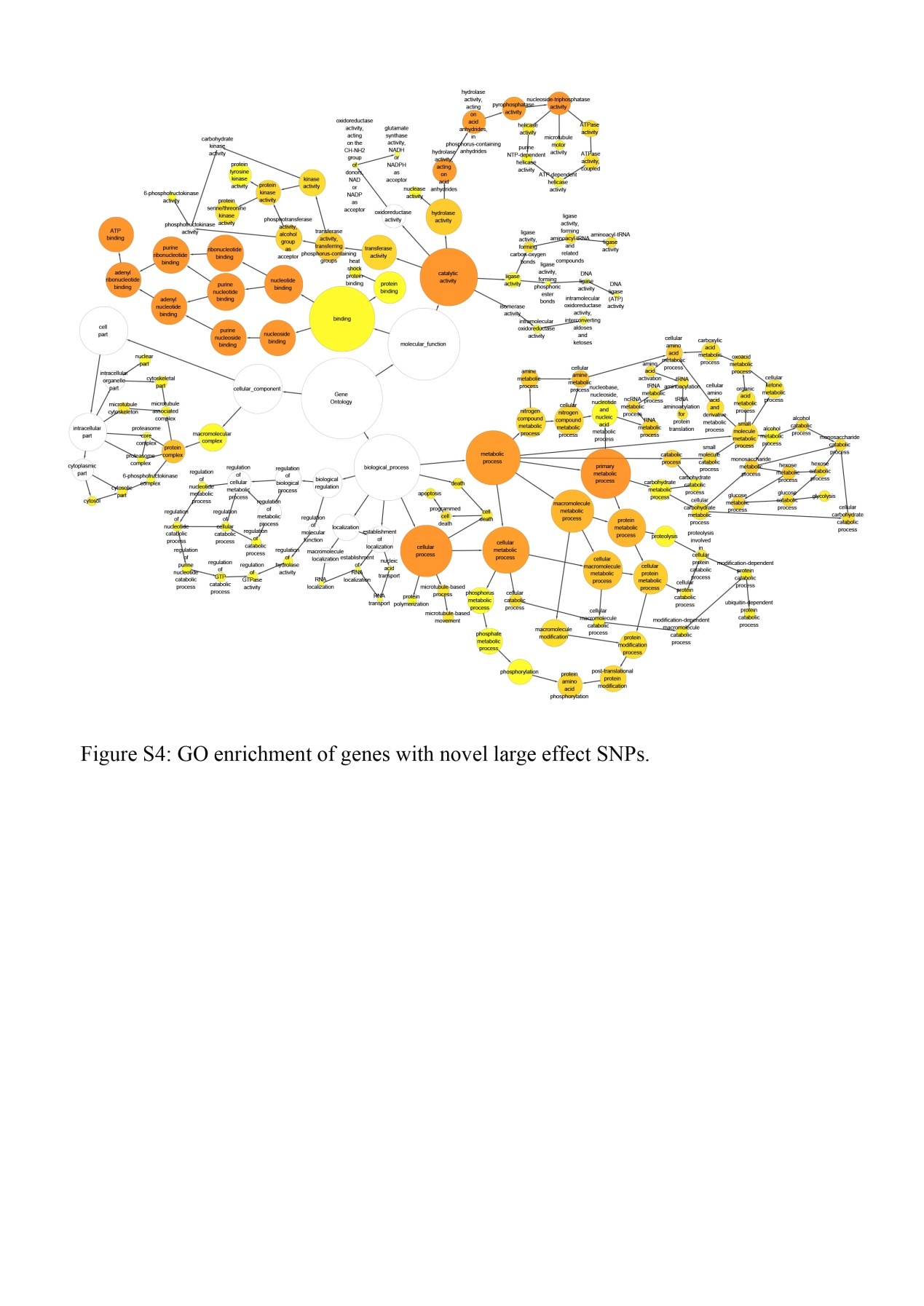

Supplement: Supplementary file 2 [file Table_1.DOCX]
